# Evolutionary analysis of ascorbate-glutathione cycle genes across green plants with lineage-specific profiling in grapevine (*Vitis vinifera* L.)

**DOI:** 10.1093/hr/uhaf247

**Published:** 2025-09-19

**Authors:** Jianxiang Liang, Menghao Xu, Bohan Yang, Jiaqi Liu, Zhizhuo Xu, Xiukun Yao, Jiang Lu, Peining Fu

**Affiliations:** Center for Viticulture and Enology, School of Agriculture and Biology, Shanghai Jiao Tong University, Shanghai 200240, China; Center for Viticulture and Enology, School of Agriculture and Biology, Shanghai Jiao Tong University, Shanghai 200240, China; Center for Viticulture and Enology, School of Agriculture and Biology, Shanghai Jiao Tong University, Shanghai 200240, China; Center for Viticulture and Enology, School of Agriculture and Biology, Shanghai Jiao Tong University, Shanghai 200240, China; Center for Viticulture and Enology, School of Agriculture and Biology, Shanghai Jiao Tong University, Shanghai 200240, China; Center for Viticulture and Enology, School of Agriculture and Biology, Shanghai Jiao Tong University, Shanghai 200240, China; Center for Viticulture and Enology, School of Agriculture and Biology, Shanghai Jiao Tong University, Shanghai 200240, China; Center for Viticulture and Enology, School of Agriculture and Biology, Shanghai Jiao Tong University, Shanghai 200240, China

## Abstract

Ascorbate-glutathione (AsA-GSH) cycle genes are vital for plant processes like stomatal regulation, nutrient uptake, and stress responses. However, the relationships between the origin and expansion of the AsA-GSH cycle genes and the adaptive evolution of land plants are still unclear. To investigate their evolutionary origins and functional differences, we first used phylogenetic and expression analyses of 2424 AsA-GSH genes (1059 *APXs*, 364 *DHARs*, 629 *MDHARs*, and 372 *GRs*) derived from 127 green plants to investigate their evolutionary history and functional divergence in green plants. The results highlighted a strong linkage between plant AsA-GSH cycle genes and their adaptation to environmental stress. In grapevine (*Vitis vinifera*), 16 AsA-GSH genes were identified and analyzed for gene structure, motifs, *cis*-regulatory elements, and transcription factors network. Gene expression profiling demonstrated their involvement in growth, hormonal regulation, and responses to biotic (*Plasmopara viticola* infection) and abiotic (cold, heat, salt, and drought) stresses. Functional validation showed that some of these grapevine genes, such as *VvAPX6*/*7*/*8*, *VvDHAR1*, *VvMDHAR2*, and *VvGR2*, are localized in diverse cellular compartments effectively mitigate oxidative stress through ROS scavenging. This study enhances our understanding of the evolutionary dynamics and functional diversification of AsA-GSH cycle genes in green plants, and the stress resilience in grapevine.

## Introduction

Plants constantly generate reactive oxygen species (ROS), including singlet oxygen (^1^O_2_), superoxide anion (O_2_^·-^), hydrogen peroxide (H_2_O_2_), and hydroxyl radicals (^•^OH) [1]under various biotic and abiotic stress conditions. These byproducts of various metabolic processes during stress could also cause oxidative damage to cells [2]. To prevent oxidative damage from ROS, plants employ various antioxidant mechanisms during both normal growth and stressful conditions [3]. Among them, the ascorbic acid (AsA)-glutathione (GSH) cycle is a key metabolic pathway for detoxifying ROS, serving as a central component of the plant antioxidant defense system by eliminating H₂O₂ from the cytoplasm and various organelles to maintain cellular redox homeostasis [4]. The pathway involves enzymes such as ascorbate peroxidase (APX), dehydroascorbate reductase (DHAR), mono-DHAR (MDHAR), and glutathione reductase (GR), along with antioxidant metabolites like AsA, GSH, and NADPH, which form redox couples with distinct redox potentials and concentrations. Maintaining redox homeostasis in plants is important for protecting them from oxidative damage [5]. Research involving mutants and transgenic plants with altered expression of enzymes or metabolites in the AsA-GSH cycle has strongly demonstrated the correlation between this pathway and stress tolerance [6]. APX is the initial enzyme in the cycle that detoxifies H_2_O_2_ by catalyzing AsA peroxidation and producing MDHA radicals. MDHA is enzymatically regenerated to AsA via MDHAR or nonenzymatically converted into both AsA and dehydroascorbic acid (DHA). DHAR then using GSH as a reducing agent reduce DHA to AsA [7]. GSH is restored from oxidized glutathione dimers through the action of NADPH-dependent GR. Then, GR restores oxidized glutathione, ensuring the maintenance of a reduced glutathione pool in the cell, and supports antioxidant activity through the active APX isoforms [8]. The AsA-GSH cycle is found in all organelles, where it detoxifies H₂O₂ and regulates the levels of AsA and GSH across various cellular compartments, protecting cells from the damaging effects of ROS induced by biotic and abiotic stresses [9].

The regulation of AsA-GSH cycle genes by transcription factors (TFs), hormonal signals, and other regulatory elements underscores their essential role in maintaining ROS balance [10]. Many studies have shown that APXs can enhance tolerance to a range of abiotic stresses [11]. For example, overexpressed chloroplastic APXs were observed to exhibit increased salt tolerance in *Jatropha curcas* [12]. Similarly, overexpressing peroxisomal APX improved salt tolerance in *Salicornia brachiata* and drought tolerance in transgenic *Arachis hypogaea* [13]. Moreover, overexpressing chloroplastic tAPX enhanced their tolerance to chilling stress under conditions of high light intensity in *Nicotiana tabacum* [14]. Five different *DHARs* present in organelles (chloroplasts or mitochondria) or in the cytoplasm have been identified in Arabidopsis [15]. Overexpression of DHAR increased AsA accumulation and conferred antioxidant and salt stress resistance in *Lycopersicon esculentum* [16]. Additionally, *DHAR* is essential for regulating plant growth and development. The absence of *DHAR* led to rapid depletion of AsA in *Oryza sativa*, resulting in a slower rate of leaf expansion and ultimately impairing overall plant growth and development [17]. For *MDHARs*, six isoforms of *MDHARs* have been identified in Arabidopsis, including two peroxisomal, two cytosolic, and one dually targeted to both chloroplasts and mitochondria [5]. The overexpression of Arabidopsis MDHARs in *N. tabacum* enhanced the transgenic plants’ tolerance to salt and drought stresses [18]. Then, studies have shown that *GR* is located mainly in chloroplasts [19], but it is also present in the cytoplasm, mitochondria and peroxisomes. In *N. tabacum*, overexpressing GRs in the chloroplasts showed higher levels of GSH and AsA, and improved tolerance to high light and chilling stress in the transgenic plants [20]. In *Gossypium hirsutum*, the overproduction of chloroplastic GRs also conduced to mitigate photoinhibition under chilling stress [21].

The AsA-GSH cycle is a central antioxidant pathway in plants, playing vital roles in maintaining cellular redox homeostasis and mitigating oxidative damage under various abiotic stresses. Although individual components of this cycle have been functionally characterized in several model plants, a systematic understanding of their evolutionary history across the plant kingdom remains limited. Comparative analyses from lower to higher plants can provide valuable insights into how the AsA-GSH cycle has diversified to support plant adaptation from aquatic to terrestrial environments. Grapevine (*Vitis vinifera* L.) is grown annually on around 7.9 million hectares worldwide and holds significant economic importance owing to its various applications, including fresh consumption, winemaking, and the production of juice and raisins [22]. As a result, there is ongoing interest in enhancing grapevine germplasm to improve berry quality, stress resistance, and cultivation efficiency. This drives research into identifying genes that regulate plant growth and development, helping to develop strategies for molecular breeding to address various environmental stresses. Although the potential functions of some AsA-GSH cycle genes have been investigated in previous studies [23], information with respect to their roles in grapevine is very limited, and further investigations are needed. In recent years, the sequencing and assembly of grapevine genomes has been gradually improved. The grapevine genome was first released in 2007 (PN40024, 8X), and that was the first sequenced fruit crop [24]. Since then, several updated versions have been made available, including the 12X.v2 assembly and its enhanced annotation VCost.v3 in 2017 [25], followed by PN40024.v4 in 2021 [26], and the more recent genome sequences (PN40024.T2T (v5)) [27] By integrating evolutionary analysis with expression profiling and functional characterization in grapevine, this study aims to uncover both conserved and specialized roles of the AsA-GSH cycle, thereby advancing our understanding of redox regulation in plants and providing candidate genes for crop improvement.

## Results

### Identification and evolutionary profiling of AsA-GSH cycle genes across plant species

**Figure 1:**
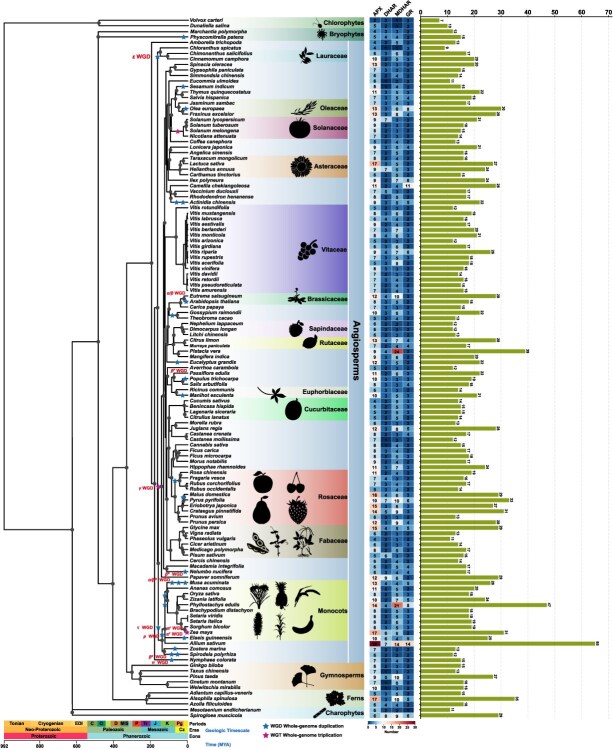
Numbers of AsA-GSH cycle genes across different plant lineages under phylogenetic tree. Red stars denote WGD events, while blue stars indicate WGT events. Plant silhouettes for representative species were obtained from PhyloPic (http://phylopic.org)

To investigate the evolutionary conservation and diversification of AsA-GSH cycle genes across different plant species, we used the *DeepGOPlus* method [28] to identify these genes in 127 plant species, representing key evolutionary lineages of the plant including 113 Angiosperms, two bryophytes, two charophytes, two chlorophytes, three ferns, and five gymnosperms. This selection allows us to trace the evolutionary trajectory of AsA-GSH cycle genes from early-diverging green algae to land plants, including both nonvascular and vascular groups. Additionally, the inclusion of a large number of angiosperms provides sufficient resolution for studying gene family expansion and functional diversification within this highly evolved clade. We identified 2424 genes (1059 *APXs*, 364 *DHARs*, 629 *MDHARs*, and 372 *GRs*) belonging to the AsA-GSH cycle gene family from 127 phylogenetically representative Viridiplantae taxa, comprehensively covering major clades spanning key embryophyte and streptophyte (Zygnemophyceae and Charophyceae), chlorophytes, ferns, and bryophytes. Most of the AsA-GSH cycle family genes reported here have never been identified before, while some of which were reclassified into different gene families (Fig. 1) (Supplementary Table S1).

**Figure 2:**
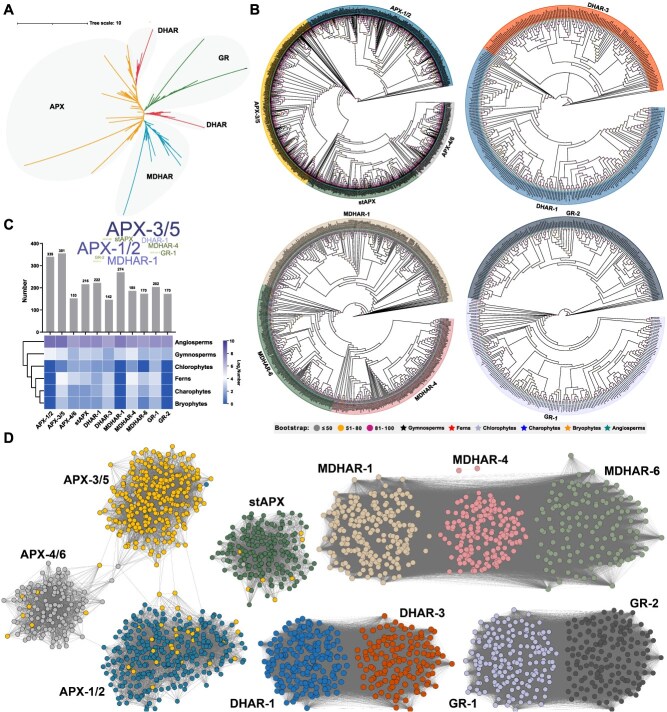
Phylogenetic classification and syntenic relationships of AsA-GSH cycle genes in plant lineages. (A) The phylogenetic tree was reconstructed for AsA-GSH cycle genes across 127 representative plant lineages, resolving these genes into four evolutionarily distinct clades with robust nodal support. (B) Phylogenetic tree for the APX, DHAR, MDHAR, and GR proteins. (C) Quantitative profiling of gene counts across functional subgroups within the AsA-GSH cycle pathway. (D) Synteny network of AsA-GSH genes based on genome-wide collinear relationships

In Chlorophytes and Charophytes, which represent early-diverging green algae [29], the number of AsA-GSH cycle genes was relatively low and conserved. *Dunaliella salina* contained five *APXs*, two *DHARs*, one *MDHARs*, and two *GRs* (total 10), while *Volvox carteri* had two *APXs*, two *DHARs*, one *MDHARs*, and two *GRs* (total 7). These species, among the earliest-diverging photosynthetic eukaryotes, exhibited the lowest gene counts, with total AsA-GSH gene numbers of 7 and 10, respectively. In Charophytes, which are phylogenetically closer to land plants, *Spirogloea muscicola* harbored six *APXs,* eight *DHARs*, six *MDHARs*, and six *GRs*, yielding a total of 29 genes, whereas *Mesotaenium endlicherianum* possessed six *APXs*, two *DHARs*, two *MDHARs*, and one *GRs* (total 11), indicating a moderate expansion compared to chlorophytes, particularly in redox recycling genes. Bryophytes retained a minimal yet complete complement of the AsA-GSH cycle. *Marchantia polymorpha* encoded four *APXs*, three *DHARs*, three *MDHARs*, and two *GRs* (total 12), whereas *Physcomitrella patens* showed a slightly higher number, with five *APXs*, four *DHARs*, four *MDHARs*, and two *GRs* (total 15). These results support a conserved architecture of the antioxidant system in early land plants. Ferns exhibited a clear increase in gene numbers. For example, *Adiantum capillus-veneris*, five *APXs*, three *DHARs*, five *MDHARs*, and two *GRs* were identified (total 15), while *Alsophila spinulosa* showed significantly expanded gene families with 17 *APXs*, 3 *DHARs*, 10 *MDHARs*, and 5 *GRs*, totaling 35 genes, which more than twice the number found in other fern species, likely a result of a lineage-specific whole-genome duplication event (Fig. 1) (Supplementary Table S1).

Among gymnosperms, *Ginkgo biloba* had eight *APXs*, two *DHARs*, three *MDHARs*, and two *GRs* (total 15), while *Pinus taeda* exhibited notable expansion in the *MDHAR* family with 10 *MDHARs*, accompanied by nine *APXs*, five *DHARs*, and three *GRs* (total 27). These results indicate moderate expansion in seed plants prior to the emergence of angiosperms. Angiosperms showed the most pronounced increase in both total gene number and family-level divergence. In monocots, most species contained between 18 and 30 AsA-GSH genes. For example, *Oryza sativa* had eight *APXs*, two *DHARs*, five *MDHARs*, and three *GRs* (total 18); *Sorghum bicolor* had eight *APXs*, three *DHARs*, five *MDHARs*, and three *GRs* (total 19); while *Zea mays* exhibited a higher gene count with 17 *APXs*, 6 *DHARs*, 6 *MDHARs*, and 2 *GRs* (total 33). *Phyllostachys edulis* showed the most extensive gene expansion in monocots, with 14 *APXs*, 4 *DHARs*, 21 *MDHARs*, and 8 *GRs*, reflecting lineage-specific amplifications likely associated with whole-genome duplication events [30]. Dicotyledonous plants exhibited the broadest range of gene family sizes and the highest total gene counts. For example, *Arabidopsis thaliana* possessed eight *APXs*, three *DHARs*, five *MDHARs*, and two *GRs* (total 18), consistent with its status as a reference genome. *Solanum lycopersicum* contained nine *APXs*, seven *DHARs*, three *MDHARs*, and two *GRs* (total 21), while *Glycine max* exhibited 15 *APXs*, 4 *DHARs*, 5 *MDHARs*, and 5 *GRs* (total 29). *Pistacia vera* showed extreme expansion in the *MDHAR* family with 24 members, along with nine *APXs*, four *DHARs*, and two *GRs* (total 39). In *Camellia chekiangoleosa*, 11 *APXs*, 2 *DHARs*, 4 *MDHARs*, and 11 *GRs* were identified, with the *GR* family showing a striking enrichment relative to other dicots. Across the angiosperms analyzed, total AsA-GSH gene counts ranged from 16 (*V. vinifera*) to 66 (*Allium sativum*), with dicots tending to show greater family-specific biases and asymmetrical expansions than monocots (Fig. 1) (Supplementary Table S1). Together, these data reveal a clear and quantifiable evolutionary trajectory of the AsA-GSH antioxidant system, from sparse and conserved gene sets in chlorophytes to highly expanded, lineage-specific repertoires in flowering plants.

### Phylogenetic profiling reveals wide and lineage-specific AsA-GSH cycle genes

To elucidate the evolutionary trajectories of phylogenetically distinct clades in the AsA-GSH cycle gene family, we reconstructed maximum likelihood (ML) phylogenies based on genomic data from 127 phylogenetically diverse plant taxa (Fig. 2A) (Supplementary Table S2). First, the *APX* gene family was divided into four subclasses (APX-1/2, APX-3/5, APX-4/6, and stAPX) based on the previous classification criteria [31]. Specifically, the APX homologs of *A. trichopoda*, which is recognized as the earliest-diverging extant angiosperm and exhibit pan-subclade distribution across all four evolutionary lineages, providing phylogenetic evidence that ancestral APX paralogs originated within the most recent common ancestor of crown angiosperms [32] (Fig. 2B). To delineate the evolutionary trajectories of the four APX clades, we systematically expanded our phylogenomic analyses to encompass key nonangiosperm embryophyte lineages, comprising five gymnosperm species (*Ginkgo biloba*, *Pinus taeda*, *Gnetum montanum*, *Taxus chinensis*, and *Welwitschia mirabilis*), three ferns (*Adiantum capillus-veneris*, *Azolla filiculoides*, and *Alsophila spinulosa*), two chlorophytes (*Dunaliella salina* and *Volvox carteri*), two charophytes (*Mesotaenium endlicherianum* and *Spirogloea muscicola*) and two bryophytes (*Marchantia polymorpha* and *Physcomitrella patens*) (Supplementary Table S1). Three subgroups (APX-3/5, APX-4/6, and stAPX) of the *APX* family were also found in these lineages (Fig. 2B) (Supplementary Table S2), suggesting that the last common ancestor of land plants already contained five distinct *APX* genes, which are retained in all five representative gymnosperm lineages. However, the presence of genes in the APX-1/2 subgroup began in gymnosperms, suggesting that gymnosperms may be the origin of the common ancestor of this subgroup. Furthermore, our phylogenetic analysis revealed a group of moss-specific genes that are located on the same branch as both subgroups APX-3/5, APX-4/6, and stAPX, implying that these subgroups and this moss-specific gene group have a common origin (Fig. 2B). The *MDHAR* and *GR* gene family have MDHAR-1 and GR-2 subgroups, which are found only in gymnosperms and angiosperms. The absence of these subtypes in earlier-diverging plant groups suggests that they originated in the lineage leading to seed plants, likely in response to their specific physiological and ecological needs.

To further understand the evolutionary relationship among the AsA-GSH proteins in transitional plants, we performed additional analyses using the APX, DHAR, MDHAR, and GR sequences of ferns, chlorophytes, charophytes, and bryophytes to gain a deeper understanding of the evolutionary relationships of AsA-GSH proteins in species within the water–land transition zone (Fig. S1). Phylogenetic reconstruction reveals three evolutionarily conserved subgroups (APX-3/5, APX-4/6, and stAPX) within APX homologs of embryophytes and streptophytic algae. Green algae (*Dunaliella salina* and *Volvox carteri*) live in aquatic environments and are subject to environmental stress in different ways, and thus may rely on other antioxidant mechanisms, with less need for cytoplasmic *APX* genes. This also reflects the difference in evolutionary adaptation between water–land transition zone plants and terrestrial plants. Specifically, each subgroup contains charophytes, bryophytes, and ferns, but the *APXs* of chlorophytes occur only in stAPX and APX-4/6, and lack APX-3/5 subgroups (Fig. S1). These findings suggest that APX-3/5 subgroups may have evolved as terrestrial plants adapted to stress conditions such as drought and photooxidation, whereas these algae do not experience the same environmental stresses and therefore do not necessarily need this subclass of genes. Notably, the MDHAR-1 and GR-2 subgroups of *MDHAR* and *GR* genes were also missed in the water–land transition zone, which may be due to a similar reason like the *APX* gene. Motif analysis further confirmed the structural conservation of APX, DHAR, MDHAR, and GR proteins, while lineage-specific variation points to functional specialization during plant evolution (Figs S2–S5).

### Conserved synteny variations of AsA-GSH cycle genes

Synteny, the preserved order of genes on chromosomes across various genomes, is an essential tool for investigating the fundamental principles of genomic organization [33]. To elucidate the evolutionary trajectory of AsA-GSH cycle genes, we developed a cross-species phylogenomic collinearity database encompassing 127 phylogenetically diverse taxa and delineated functionally conserved gene clusters within this network. Phylogenomic analysis of APXs, DHARs, MDHARs, and GRs was performed by integrating phylogenetic and linkage relationships. Analysis of 2424 AsA-GSH cycle genes (comprising 1059 *APXs*, 364 *DHARs*, 629 *MDHARs*, and 372 *GRs*) identified 389 709, 94 999, 273 400, and 112 476 syntenic associations across these functional categories, respectively, through genome-wide collinearity scanning. The presence of strong syntenic signals among homologous gene pairs within each major clade further validates the classification of AsA-GSH cycle genes in angiosperms derived from phylogenetic analysis. Among them, the APX synteny network revealed four main clusters, MDHAR revealed three major clusters, and DHAR and GR revealed two major clusters each (Fig. 2D). Notably, in the APX synteny network, APX-1/2 subgroup genes showed weak synteny with other clusters (Fig. 2D). Phylogenetic reconstruction demonstrated sister-clade relationships between APX-1/2 and APX-3/5 subgroups, while APX-4/6 subgroup formed an evolutionarily divergent branch. Comparative macro-collinearity analysis across basal angiosperms (*Amborella trichopoda*, *Papaver somniferum*, and *Nymphaea colorata*) revealed ancestral whole-genome duplication events preceding the radiation of APX-1/2, APX-3/5, APX-4/6, and stAPX clades. APX-3/5 subgroup dominated collinear clusters constituted >50% of network topology (Fig. 2C–D), with taxon-specific node distribution patterns emerging from phylogenomic reconciliation. Some nodes of this cluster interacted and merged with other clusters, which contained genes from ferns, bryophytes, charophytes, basal angiosperms, and eudicots. Notably, *Spirogloea muscicola SmAPX1/4*, *Cinnamomum camphora CcAPX2*, *Rhododendron henanense RhAPX3* and *Cannabis sativa CsAPX8* became bridges connecting APX-1/2 and APX-3/5 subgroups; *Jasminum sambac JsAPX1* and *Taraxacum mongolicum TmAPX4* became bridges connecting APX-1/2 and APX-4/6 subgroups; and *Arabidopsis thaliana AtAPX4* and *Dunaliella salina DsAPX2/4* became bridges connecting APX-1/2 and APX-3/5 subgroups. These ‘bridge’ genes may represent the intermediate states of the *APX* gene family transitioning from one subfamily to another during evolution, indicating that these genes have more extensive homology between different subfamilies and that these genes connecting different subfamilies may have unique or broad functions.

### Whole-genome characterization of AsA-GSH cycle genes in grapevine

Following comprehensive analysis of the evolutionary history of AsA-GSH cycle genes across green plants, we observed lineage-specific expansion patterns and functional diversification, particularly in vascular plants and angiosperms. To further illustrate how these broad evolutionary patterns translate into finer-scale functional diversification within specific plant lineages, we subsequently focused on grapevine (*V. vinifera*), the core eudicot with rich sequenced high-quality genomes, transcriptomic resources, and well-established genetic transformation systems. To explore the evolutionary and functional diversity of the AsA-GSH cycle gene family in grape species, we performed a genome-wide survey in 16 representative *Vitis* species. While the domesticated grapevine (*V. vinifera*) encoded 16 genes, several wild North American species, including *V. riparia* (26 genes), *V. monticola* (21 genes), and *V. berlanderi* (20 genes), exhibited considerably larger gene families, particularly evident in *MDHARs* and *GRs* (Fig. 1) (Supplementary Table S1). These findings suggest that *V. vinifera* underwent gene loss or contraction events during domestication and selective breeding, leading to a reduced diversity of antioxidant related genes relative to its wild counterparts. Such gene loss likely contributes to the lower adaptability of cultivated grapevines to abiotic stresses compared to wild species, which retain broader stress-tolerance capacities shaped by selective pressures in their native habitats. Moreover, wild *Vitis* species typically distributed to environments with more severe abiotic challenges, such as drought, cold, and oxidative stress, and often demonstrate enhanced environmental resilience relative to *V. vinifera* [34]. Thus, the greater retention of certain AsA-GSH cycle genes observed in wild grapevines likely reflects natural selection favoring enhanced ROS scavenging capacities.

**Figure 3:**
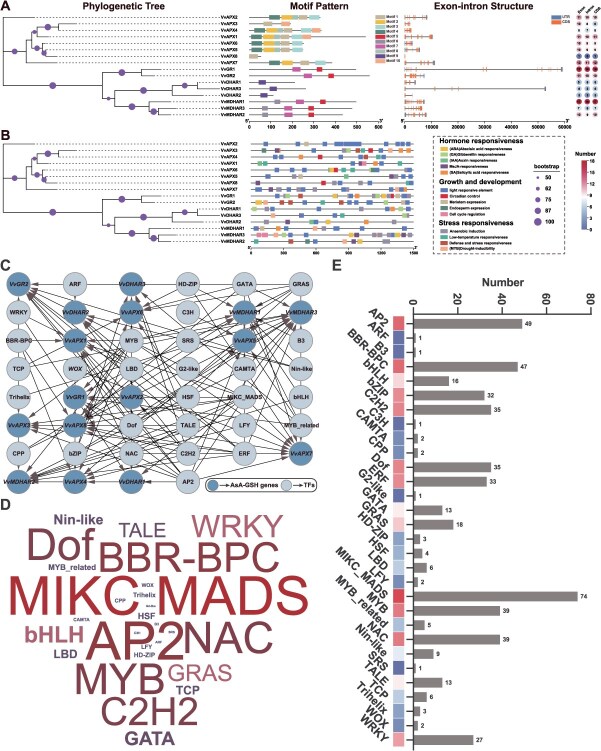
Phylogenetic relationship, gene structure, CREs and TFs regulatory network of grapevine AsA-GSH cycle genes. (A) The gene structures of *VvAPXs*, *VvDHARs*, *VvMDHARs*, and *VvGRs* are shown, and the scale bar indicates 1 kb. (B) The distribution of CREs within the 2000-bp upstream promoter region of *VvAPXs*, *VvDHARs*, *VvMDHARs*, and *VvGRs*. (C) Network analysis showing relationships between putative TFs (lightsteelblue nodes) and grapevine AsA-GSH cycle genes (steelblue nodes). TF families were classified according to the Plant TF Database. (D) Word cloud for TFs. (E) Statistical distribution of TFs

A total of 16 AsA-GSH cycle genes (eight *VvAPXs*, three *VvDHARs*, three *VvMDHARs*, and two *VvGRs*) were identified in the grapevine (*V. vinifera*) genome, and named according to their chromosomal positions (Supplementary Table S3). These proteins ranged from 55 aa (VvAPX8) to 559 aa (VvGR2) in length, with molecular weights from 6.13 to 60.05 kDa and pIs from 4.93 (VvDHAR2) to 9.51 (VvAPX3) (Fig. S6). Most proteins were predicted to be stable *in vitro*, hydrophilic (negative GRAVY), and enzymatically active (Supplementary Table S3). Most grapevine AsA-GSH cycle proteins are predicted to localize in the nucleus, plastid, or cytoplasm, while a few, including VvAPX3, VvMDHAR1/2/3, and VvDHAR1, while a few others may localize to mitochondria, peroxisomes, or chloroplasts, suggesting diverse potential biological functions. Structural and motif comparisons revealed subgroup-specific patterns[35]. *VvAPX2/5/6* (APX-1/2 and APX-4/6 subgroups) share motifs 1, 4, and 8. *VvAPX1/3/4/8* (APX-3/5 subgroup) mainly retain motif 1, whereas *VvAPX7* (stAPX subgroup) contains motifs 1–4. Notably, VvAPX3/8 lack several conserved motifs. This may be due to the generation of new genes during the evolution due to gene duplication events or gene annotation, resulting in a shorter open reading frame (ORF) length [36]. The relative numbers of coding sequences (CDSs) in the 16 grapevine AsA-GSH cycle genes ranged from 1 to 17 (Fig. 3A). Among them, *VvMDHAR1* exhibits the highest number of transcript variants with 17 CDSs, followed by *VvGR1* (16 CDSs), *VvAPX7* (12 CDSs), and *VvAPX1* (11 CDSs), suggesting that these genes may undergo complex alternative splicing [37][38].

*Cis*-regulatory elements (CREs) are located in the promoter regions of genes and are essential for gene expression, significantly impacting plant growth and the response to stress [39]. the CREs in the promoters of grapevine AsA-GSH cycle genes were investigated by analyzing the 2000-bp region upstream of their transcriptional start sites using the PlantCARE database. A total of 234 CREs were identified in the promoter regions of AsA-GSH cycle genes (Supplementary Table S4), and 14 representative CREs are shown (Fig. 3B). Hormone-related elements (69 CREs) are mainly responsive to MeJA (24, 34.78%) and ABA (21, 30.43%), with additional motifs for GA (10, 14.49%), SA (11, 15.94%), and IAA (3, 4.35%). These elements are particularly abundant in *VvAPX6*, *VvDHAR1*, and *VvMDHAR2*, suggesting that these genes are strongly regulated by hormones. Growth and development elements (108 CREs) are mainly light-responsive, with additional motifs related to circadian rhythm, meristem, endosperm, and cell cycle regulation. Stress responsiveness elements (57 CREs) are largely associated with anaerobic induction, with LTR and TC-rich repeats enriched in *VvMDHARs* and *VvGRs*. Moreover, potential transcriptional regulatory factors of grapevine AsA-GSH cycle genes were identified by PlantTFDB [40] and visualized by Cytoscape (Fig. 3C) (Supplementary Table S5). A total of 519 TFs from 30 different TF families were identified, including those well-known TFs such as MYB, NAC, APX2, ERF, bZIP, WRKY, etc. (Fig. 3D). Among them, *VvAPX8* interacted with 86 TFs, whereas *VvDHAR3* was controlled by only 10. Such diversity in TFs interactions highlights the integration of AsA-GSH cycle genes into multiple signaling and developmental pathways, reinforcing their central role in grapevine growth, stress adaptation, and redox regulation. In conclusion, the marked divergence in gene structure, motifs composition, CREs distribution, and TFs regulation highlights the evolutionary diversification of grapevine AsA-GSH cycle genes and suggests that different subgroups have undergone specialized functional adaptation to balance growth, hormone response, and stress tolerance.

### Collinearity analysis of grapevine AsA-GSH cycle genes

**Figure 4:**
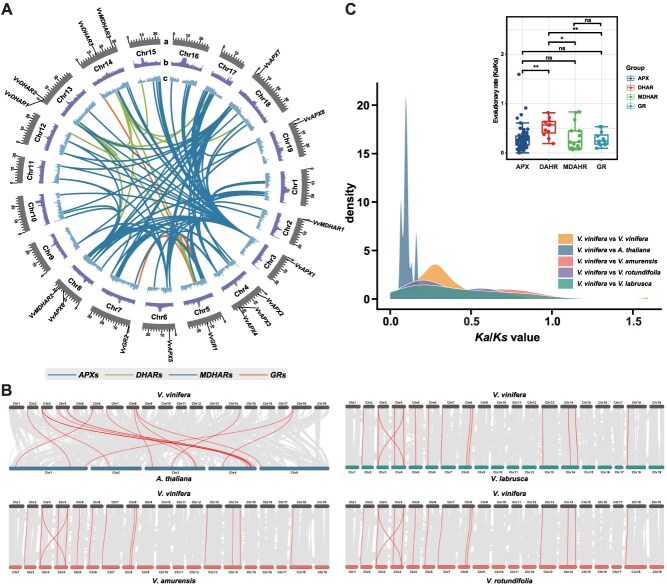
Chromosomal distribution and gene synteny. (A) Distribution and collinearity of AsA-GSH cycle genes in the grapevine genome. Panels: (a) 19 chromosomes with mega base units; (b) GC content; (c) Gene density. (B) Synteny analysis of AsA-GSH cycle genes in European (*V. vinifera*), East Asian (*V. amurensis*), Muscadinia (*V. rotundifolia*), and American (*V. labrusca*) species, with red lines highlighting syntenic gene pairs. (C) Histogram of *Ka*/*Ks* ratios for AsA-GSH cycle genes

The chromosomal distributions of the grapevine AsA-GSH cycle genes were analyzed (Fig. 4A). Gene collinearity analysis conducted via MCScanX in grapevine identified 117 duplication events, predominantly consisting of segmental duplications (*n* = 115) with limited tandem duplication occurrence (*n* = 2), indicating that segmental duplication was the major force driving gene family expansion. We calculated the nonsynonymous substitution (*Ka*) and synonymous substitution (*Ks*) ratio for all 117 gene pairs, most gene pairs *Ka*/*Ks* < 1 suggesting strong purifying selection and functional conservation, with divergence times estimated from 0.08 to 218.46 million years ago (MYA) (Supplementary Table S6). Comparative collinearity analysis between the grapevine AsA-GSH cycle genes and corresponding genes from a model plant species (*A. thaliana*) and three geographically representative species of grapevine (*V. amurensis*, *V. labrusca*, and *V. rotundifolia*) (Figs 4B and Fig. S7) (Supplementary Table S7). We compared the chromosome homology of *V. vinifera* with those of *A. thaliana*, *V. amurensis*, *V. labrusca*, and *V. rotundifolia* by whole genome alignment and identified 13 955, 26 604, 29 960, and 29 041 alignment blocks, respectively (Fig. 4B). For *VvAPX1/3/5/6/7*, *VvMDHAR1/2/3,* and *VvGR2* in European grapevine, the collinear relationship was observed with the corresponding genes in *A. thaliana*, *V. amurensis*, *V. labrusca* and *V. rotundifolia*, which may indicate that these genes may have conserved evolutionary functions. *Ka*/*Ks* ratios for each gene pair were calculated to examine the evolutionary pressures acting on the grapevine AsA-GSH cycle genes. The results indicated that in *Vitis* plants, such as *V. amurensis*, *V. labrusca*, and *V. rotundifolia*, *Ka*/*Ks* analysis revealed that all collinear gene pairs are under purifying selection, as evidenced by ratios consistently below 1. Moreover, in the comparison of plants such as *A. thaliana*, the *Ka*/*Ks* ratios were also mainly less than 1, and only two gene pairs could not be calculated. This might be attributed to the high level of synonymous mutations at many sites in the DNA sequences, suggesting significant sequence divergence and considerable evolutionary distance between these two genes in both plants.

### AsA-GSH cycle gene expression in different grapevine tissues

**Figure 5:**
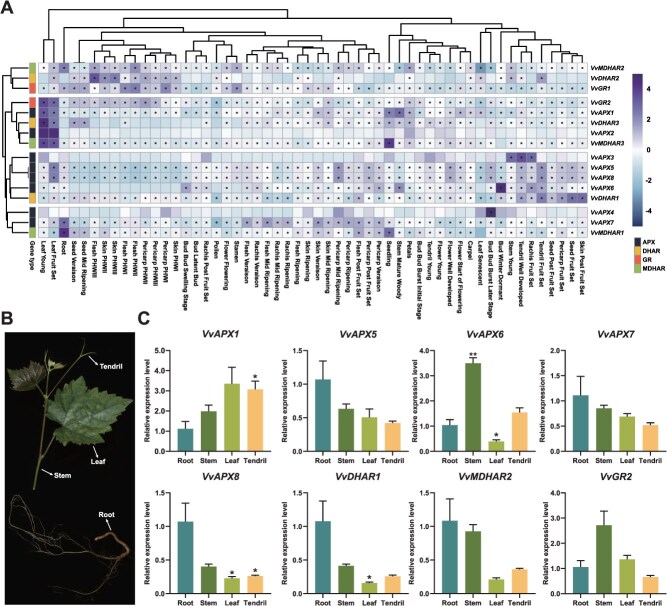
Expression profiles of the grapevine AsA-GSH cycle genes in different tissues. (A) Transcriptome-based expression analysis of AsA-GSH cycle genes in 54 tissues and developmental stages of *V. vinifera* ‘Cabernet Sauvignon’. Heatmap created from log_2_(FPKM +0.01) values, with genes marked as abundant (FPKM >50) indicated by ‘*’. (B) Tissue sampling locations for *V. vinifera* cv ‘Cabernet Sauvignon’. (C) RT-qPCR analysis of eight genes (*VvAPX1*/*5*/*6*/*7*/*8*, *VvDHAR1*, *VvMDHAR2*, and *VvGR2*) in root, stem, leaf, and tendril tissues. All experiments were independently repeated at least three times. Error bars represent standard deviation; asterisks denote significant differences compared to root (^*^P <0.05, ^**^P <0.01, ^***^P <0.001)

A comprehensive transcriptomic atlas covering 54 distinct tissues of *V. vinifera* cv. ‘Cabernet Sauvignon’ [41] was employed to examine the spatial and temporal expression dynamics of AsA-GSH cycle genes (Supplementary Table S8). *VvAPX5/7/8*, *VvDHAR1*, *VvMDHAR1/2/3*, and *VvGR1/2* were broadly expressed across developmental stages, suggesting roles in general growth and development. *VvAPX1/3/5* were highly expressed during fruit development, indicating involvement in oxidative stress management, while *VvDHAR1* and *VvGR1* showed elevated expression in young leaves and developing seeds, reflecting functions in maintaining redox balance during rapid growth. *VvAPX6/7* were predominantly expressed in mature woody stems, potentially contributing to lignification and stress responses. Subfamily members exhibited variable expression such as *VvAPX3* and *VvAPX8* belonging to the APX-3/5 subgroup showed complementary patterns, with *VvAPX3* enriched in tendrils, young stems, and dormant buds, while *VvAPX8* increased across most tissues during development. Similarly, *VvAPX1/2*, *VvDHAR3*, *VvMDHAR3*, and *VvGR2* were mainly expressed in leaf-young and leaf–fruit set stages, whereas *VvAPX7* and *VvMDHAR1* were highly expressed mainly in the root (Fig. 5A). Meanwhile, quantitative real-time PCR (RT-qPCR) was conducted to examine the expression levels of all AsA-GSH cycle genes in different grapevine tissues (root, tendril, stem, and leaf) under normal physiological conditions (Fig. 5B–C). Genes associated with the grapevine AsA-GSH cycle genes were found across various tissues, exhibiting unique yet somewhat overlapping expression patterns. In comparison with expression levels in roots, *VvAPX1* and *VvAPX6* in were highly expressed in tendrils and stems indicating that important antioxidant roles played in these tissues. In contract, *VvAPX8* and *VvDHAR1* are predominantly expressed in roots, suggesting their potential involvement in the antioxidant defense system in root tissues. *VvAPX5*/*7* and *VvGR2* exhibit expression across multiple tissues, with notably higher levels in roots and stems, indicating their broader roles in oxidative stress response in these organs.

### Multihormone regulation of AsA-GSH cycle genes in grapevine

**Figure 6:**
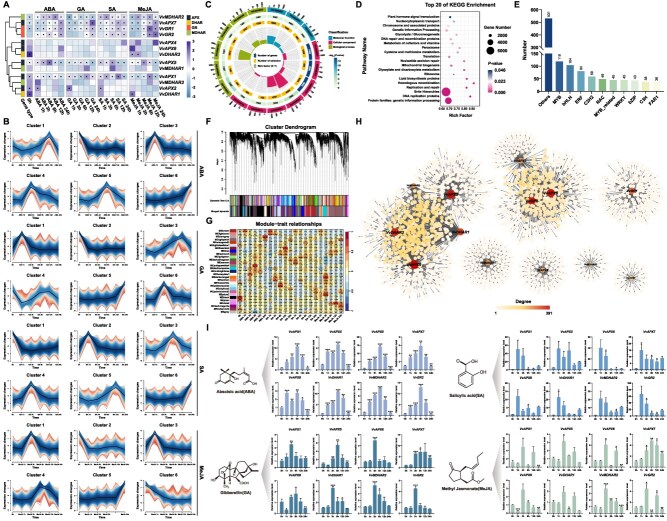
Expression profiles of the grapevine AsA-GSH cycle genes in hormone treatments (ABA, GA, SA and MeJA). (A) Hierarchical clustering of gene expression profiles in hormone treatments. Heatmap based on log_2_(FPKM +0.01) values, with genes marked as abundant (FPKM >50) indicated by ‘*’. (B) Mfuzz clustering of gene expression during hormone treatments in grapevine. (C) Gene enrichment from GO analysis of positively correlated Mfuzz clusters. (D) KEGG pathway enrichment of genes in positively correlated Mfuzz clusters. (E) TF regulatory network for AsA-GSH cycle genes based on Mfuzz clusters, classified by the Plant TF Database. (F) Consensus network of co-expression modules. (G) Consensus network modules exhibited correlations with tissue color, as indicated by the correlation coefficient and *P*-value. (H) Network analysis of genes in modules correlated with hormone treatments. (I) RT-qPCR analysis of 8 genes (*VvAPX1*/*5*/*6*/*7*/*8*, *VvDHAR1*, *VvMDHAR2*, and *VvGR2*) under hormone treatments. All experiments were independently repeated at least three times. Error bars represent standard deviation; asterisks denote significant differences (^*^P <0.05, ^**^P <0.01, ^***^P <0.001)

The expression profiles of the16 grapevine AsA-GSH cycle genes were analyzed at different time points after treating with four hormones (ABA, GA, SA, and MeJA). After eliminating genes with low expression levels, expression of 13 genes were analyzed (Fig. 6A) (Supplementary Table S9). *VvAPX1/5/7*, *VvMDHAR2,* and *VvGR1* were highly expressed at different time points after treated with these four hormones. Among them, most AsA-GSH cycle genes showed early induction following ABA and SA application, particularly within the first 1–3 h. Genes such as *VvAPX1/5* and *VvMDHAR1* were strongly upregulated at 1 h under ABA treatment, suggesting a rapid antioxidant response. However, this induction gradually weakened over time, with much lower expression observed at 12 and 24 h, indicating a transient response. GA had a relatively weaker effect, with only a few genes (e.g. *VvAPX7* and *VvGR1*) showing moderate induction, specifically at early time points. In contrast, SA triggered early activation of several *APX* genes (e.g. *VvAPX1* and *VvAPX2*), and some genes (*VvAPX1/5/7*, *VvMDHAR2*, and *VvGR1/2*) maintained elevated expression levels even at 24 h, suggesting a more sustained response. Notably, MeJA treatment resulted in strong and persistent upregulation across multiple genes throughout the 24 h period, especially *VvAPX5, VvGR2,* and *VvMDHAR2*. Mfuzz-based time-series expression analysis classified the genes into six clusters according to their expression profiles across hormone treatment stages (Fig. 6B) (Supplementary Table S10). Grapevine AsA-GSH cycle genes showed hormone-specific temporal responses ABA and GA generally triggered early induction followed by decline, while MeJA led to sustained upregulation in key clusters. GO and KEGG enrichment analyses revealed significant involvement in photosynthesis, energy metabolism, and cellular homeostasis, with key pathways related to plant hormone signaling and metabolic regulation (Fig. 6C–D) (Supplementary Table S11). PlantTFDB-based prediction identified 1149 TFs (MYB, bHLH, ERF, etc.) potentially governing stress responses and hormonal signaling (Fig. 6E) (Supplementary Table S12). WGCNA analysis revealed 29 co-expression modules, with 13 AsA-GSH cycle genes distributed across six hormone-responsive modules (Fig. 6F–H) (Supplementary Table S13). Module dynamics showed time-dependent correlations such as MElightpink4 positively correlated with ABA at 12 h (*r* = 0.59), while MEfirebrick4 negatively correlated with ABA at 1 h (*r* = −0.75). Temporal shifts were observed in MEgreenyellow’s transition from positive (1 h) to negative (12 h) correlation with MeJA. Cross-hormone correlations emerged in modules like MElightgreen, associated with both GA (1 h) and SA (12 h). Notably, MEdarkred maintained strong MeJA correlations, suggesting jasmonate pathway involvement.

Based on bioinformatics analyses and transcriptome data, eight representative AsA-GSH cycle genes were selected for RT-qPCR analysis to validate their potential roles in grapevine phytohormone response (Fig. 6I). After ABA treatment, *VvAPX1/6/7*, *VvDHAR1*, *VvMDHAR2*, and *VvGR2* were significantly upregulated between 1 and 6 h, suggesting these genes play a key role in the grapevine’s response to ABA. In contrast, response to GA is weaker, even though *VvAPX5/6/7*, *VvDHAR1*, *VvMDHAR2*, and *VvGR2* showed moderate expression changes, indicating that GA has a less impact on these genes compared to ABA. All genes were upregulated by SA, peaking between 1–6 h and significantly decreasing thereafter. Similarly, MeJA also significantly upregulated *VvAPX5/6/7*, *VvMDHAR2*, and *VvGR2* prior to 6 h. Overall, these results show that AsA-GSH cycle genes in grapevine are regulated by ABA, GA, SA, and MeJA, respectively. They are particularly strong response to ABA and SA, indicating their potential role in stress responses and plant defense mechanisms [42].

### AsA-GSH cycle gene expression under biotic and abiotic stresses in grapevine

**Figure 7:**
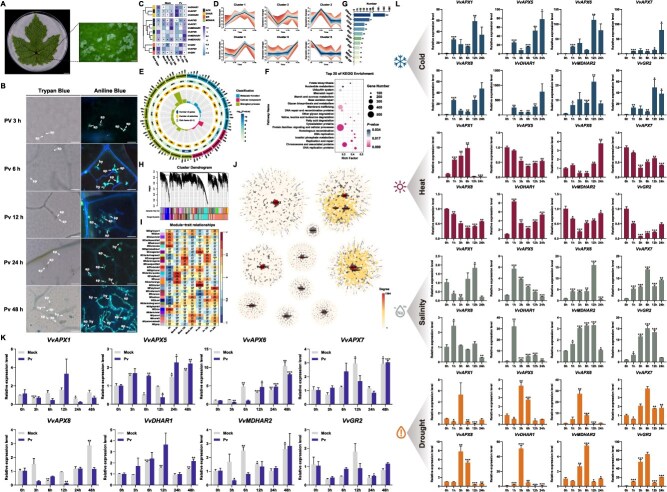
Expression of grapevine AsA-GSH cycle genes under biotic and abiotic stresses. (A) Phenotype of grapevine leaves 48 h postinoculation (hpi) with *P. viticola* zoospores (scale bar = 3 mm). (B) Growth of *P. viticola* in ‘Cabernet Sauvignon’ leaves at various time points, visualized by trypan, aniline blue staining, and UV microscopy (scale bar = 100 μm), hy, hyphae; sp, sporangia. (C) Hierarchical clustering of AsA-GSH cycle gene expression during *P. viticola* infection, with abundant genes (FPKM >50) marked by ‘*’. (D) Mfuzz clustering of gene expression during *P. viticola* infection. (E) GO analysis of positively correlated Mfuzz clusters. (F) KEGG pathway enrichment for positively correlated Mfuzz clusters. (G) TFs regulatory network for AsA-GSH cycle genes in Mfuzz clusters. (H) Co-expression modules identified by dynamic tree cut method (hierarchical cluster tree). (I) Consensus network modules correlated with tissue color, with correlation coefficients and *P*-values. (J) Network analysis of genes in modules correlated with *P. viticola* infection. (K) RT-qPCR analysis of 8 genes during *P. viticola* infection in ‘Cabernet Sauvignon’ leaves. (L) RT-qPCR analysis of 8 genes under cold (4°C), heat (45°C), NaCl, and drought treatments. Error bars represent standard deviation; asterisks indicate significant differences compared to blank control (0 h 0 mM) (^*^P <0.05, ^**^P <0.01, ^***^P <0.001)

Grapevine downy mildew (*Plasmopara viticola*) is a major disease affecting viticulture [43,44]. To understand how grapevines become infected with *P. viticola*, we observed infection of the pathogen in leaf tissue. The leaves of the ‘Thompson Seedless’ grapevine were inoculated with *P. viticola* (Fig. 7A), and the colonization of *P. viticola* within the leaf tissues was observed through trypan blue and aniline blue staining at different hours postinoculation (hpi) (Fig. 7B). These results showed that zoospores were released from sporangium and moved towards stomata within 3 hpi after inoculation. Following germination, zoospores produce a germ tube that penetrates the stomata, reaching the substomatal cavity and underlying parenchyma. By 6 hpi, the primary hyphae extend into the intercellular spaces of the mesophyll and develop haustoria. At 48 hpi, the hyphae extensively colonize the leaf parenchyma, enabling the pathogen to acquire nutrients via haustoria, suppress host defense responses, and manipulate host metabolism for its own benefit (Fig. 7B).

**Figure 8:**
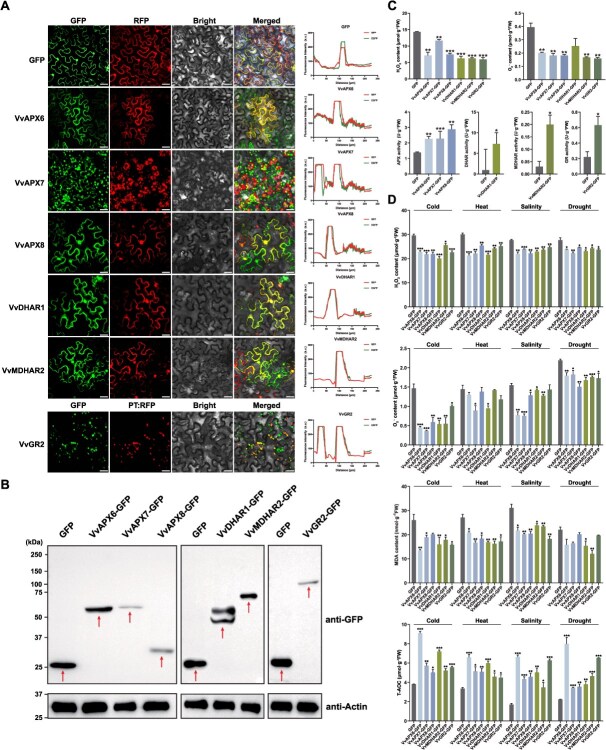
Overexpression of grapevine AsA-GSH cycle genes enhances abiotic tolerance in plants. (A) Subcellular localization of heterologous expressed GFP, VvAPX6/7/8, VvDHAR1, VvMDHAR2, and VvGR2 in leaves of *N. benthamiana* leaves. GFP and RFP were used as the control. The GFP, RFP and chloroplast autofluorescence signals were captured by laser confocal fluorescence microscopy at emission wavelengths of 488, 520, and 680 nm, respectively. Scale bar: 100 μm. (B) Western blot of total proteins from *N. benthamiana* leaves infiltrated with Agrobacterium carrying *35S:: VvAPX6*/*7*/*8*, *VvDHAR1*, *VvMDHAR2*, or *VvGR2-GFP* at 48 h postinfiltration. Red arrows indicate the fusion proteins. (C) Enzyme activity assays of APX, DHAR, MDHAR, and GR in vitro for VvAPX6/7/8, VvDHAR1, VvMDHAR2, and VvGR2-GFP proteins. (D) Physiological index determination under abiotic stress. All experiments were performed at least three times. The error bars represent the standard deviation, and asterisks highlight significant differences compared to the GFP control (^*^P <0.05, ^**^P <0.01, ^***^P <0.001)

In order to investigate the functional role of AsA-GSH cycle genes in responses to *P. viticola* infection, *P. viticola* inoculated grapevine leaves were collected from 0, 1, 3, 6, 12, and 24 hpi. During the time period, expressions of 12 AsA-GSH cycle genes were significantly changed (Fig. 7C) (Supplementary Table S9). Among them, *VvAPX1*/*5*/*7* and *VvMDHAR1*/*3* were upregulated, especially at 12 and 24 h, while *VvDHAR1*/*3* were strongly induced at all time points. These results indicate that APX, DHAR, and MDHAR contribute to ROS detoxification and redox homeostasis during pathogen stress. Temporal expression clustering using Mfuzz grouped these genes into six clusters based on their expression dynamics (Fig. 7D) (Supplementary Table S10). Cluster 5 (*VvAPX4*/*5* and *VvMDHAR1*/*2*/*3*) was induced at 24 h, suggesting a role in late defense, while Cluster 2 (*VvAPX1*/*3*/*7* and *VvGR1*/*2*) showed early downregulation at 6 h followed by partial recovery. GO and KEGG analysis revealed enrichment in pathways related to signaling, DNA replication, chromosome-associated proteins, and membrane trafficking, indicating roles in stress response, DNA repair, and cell cycle regulation (Fig. 7E and F). A predicted transcriptional network identified 451 TFs (e.g. bHLH, MYB, NAC, bZIP, etc.), as potential regulators (Fig. 7G) (Supplementary Table S12). WGCNA analysis grouped genes into 32 coexpression modules, with the 13 AsA-GSH cycle genes distributed across 10 modules, including MEblue and MEturquoise, enriched for stress-responsive genes (Fig. 7H–J) (Supplementary Table S13).

To investigate the roles of AsA-GSH cycle genes in grapevine responses to *P. viticola*, eight representative genes were selected for RT-qPCR validation. Their expression patterns showed dynamic temporal changes, indicating a multiphase defense response (Fig. 7K). Genes such as *VvAPX1/5/6/7* were strongly upregulated between 3- and 12-h postinoculation, suggesting their involvement in the early immune response. Additionally, under abiotic stress conditions (cold, heat, salinity, and drought), most genes exhibited significant and stress-specific expression changes (Fig. 7L). Under cold stress, all tested genes were strongly induced, particularly at 6 h and 12 h, reflecting enhanced antioxidant activity. Heat stress primarily affected *VvDHAR1*, *VvMDHAR2*, and *VvGR2* during early stages (0–6 h), with expression declining or stabilizing later, indicating potential heat-induced suppression. Salt treatment triggered rapid upregulation of *VvAPX5/6/7/8*, *VvDHAR1*, *VvMDHAR2*, and *VvGR2*, peaking in early time points, which may enhance ROS scavenging under salt stress. Similarly, drought stress led to early induction of most genes, particularly within the first 6 h, highlighting the key role of these genes in oxidative stress mitigation during water deficit.

### Overexpression of grapevine AsA-GSH cycle genes enhances abiotic tolerance in tobacco

To verify the subcellular localizations, we selected six potential antioxidant-associated grapevine AsA-GSH cycle genes (*VvAPX6/7/8*, *VvDHAR1*, *VvMDHAR2*, and *VvGR2*) using *N. benthamiana* leaves for the experiment. Fluorescence imaging demonstrated that the VvAPX6/8, VvDHAR1, and VvMDHAR2 proteins were distributed across the plasma membrane, cytoplasm, and nucleus, while VvAPX7 and VvGR2 proteins are localized to chloroplasts and plastids (Fig. 8A). To further evaluate their ability to scavenge H_2_O_2_, we transiently expressed these gene proteins in *N. benthamiana* leaves, with GFP as a negative control. Their post transcription expression levels were detected in *N. benthamiana* leaves by western blotting, indicating successful transient expression (Fig. 8B). Notably, the VvDHAR1-GFP showed two bands, which may be caused by post-translational modification of the protein, such as phosphorylation and glycosylation. In the physiological indexes and enzyme activity assays (Fig. 8C), we found that the contents of H₂O₂ and O₂^·-^ in all treatment groups were significantly reduced in comparison to the GFP control group, especially in VvAPX8-GFP, VvDHAR1-GFP, VvMDHAR2-GFP, and VvGR2-GFP.

To evaluate the functional role of *VvAPX6/7/8, VvDHAR1, VvMDHAR2, and VvGR2* in abiotic stress response, transient expression assays were conducted in *N. benthamiana*, followed by quantification of key physiological stress markers under cold, heat, salt, and drought treatments. Plants overexpressing any of these six gene’s proteins exhibited significantly lower levels of ROS (H₂O₂ and O₂^·-^) and malondialdehyde (MDA), while significantly increased total antioxidant capacity (T-AOC) levels were significantly increased (Fig. 8D). These results suggest that overexpression of these enzymes may help to alleviate the oxidative stress of plants and enhance their antioxidant defense capacity by reducing the accumulation of H₂O₂ and free radicals.

## Discussion

The AsA-GSH cycle is a central antioxidant pathway that regulates ROS and contributes to plant stress tolerance [6]. However, the evolutionary origins and diversification of its core genes, including *APXs*, *DHARs*, *MDHARs*, and *GRs* are poorly understood, particularly in eudicots. Our phylogenetic analyses trace these gene families back to early land plants and reveal lineage-specific expansions and neofunctionalization during spermatophyte evolution. Grapevine (*V. vinifera*) is one of the most important fruit crops worldwide. Its growth and productivity are significantly affected by environmental conditions and pest pressures. This sensitivity underscores the importance of investigating stress-responsive gene networks, such as the AsA-GSH cycle, which may offer critical insights into further understanding the mechanism of grapevine growth, hormone regulation, and stress resilience.

### Evolutionary origins and trends of AsA-GSH cycle genes

Precise identification of functional genes is key to understanding plant gene functions. Using DeepGOPlus-based prediction and phylogenetic reconstruction, we identified 2424 AsA-GSH cycle genes from 127 species spanning six major clades of green plants (Fig. 1). Early-diverging lineages, such as chlorophytes, charophytes, and bryophytes, contained relatively few and conserved members, reflecting strong purifying selection on core antioxidant functions. By contrast, vascular plants displayed more dynamic patterns. Ferns and gymnosperms showed lineage-specific expansions, likely associated with whole-genome duplications and adaptation to terrestrial environments characterized by high light, drought, or cold. For example, conifers and ginkgo maintained multiple *APXs* and *MDHARs*, consistent with long-term survival under harsh ecological conditions. In angiosperms, gene copy numbers varied widely, shaped by genome history and ecological strategies. Some species, such as garlic (*A. sativum*) and pistachio (*Pistacia vera*), exhibited substantial expansions, which may be linked to genome duplication events and specialized metabolism. These findings highlight that, while *DHAR* genes remained highly conserved across land plants, *APX*, *MDHAR*, and *GR* gene families underwent repeated diversification, reflecting both functional innovation and ecological adaptation.

To trace the evolutionary origins of the AsA-GSH cycle gene family, we constructed phylogenetic trees for 127 plant species, representing key transitions from aquatic to terrestrial environments (Fig. 2B). The *APX* gene family was divided into four subgroups (APX-1/2, APX-3/5, APX-4/6, stAPX) [31], with *Amborella trichopoda* showing genes from all subgroups, indicating their origin in the common ancestor of angiosperms. Gymnosperms were identified as the source of the APX-1/2 subgroup, while APX-3/5, APX-4/6, and stAPX subgroups were present across all the plant lineages, suggesting early evolution in land plants. The absence of APX-1/2 in early-diverging plants suggests its adaptation to terrestrial stresses like photooxidation and drought. The *MDHAR* and *GR* gene families, which are found only in gymnosperms and angiosperms, likely evolved in response to the more complex environmental challenges of land[45]. Collectively, conserved genes appearing in early-diverging lineages indicate their core roles in redox homeostasis, while those lineage- and species-specific expansions in vascular plants suggest they were derived from environmental pressures, genome duplications, and increased metabolic complexity.

### Lineage-specific duplication and rapid expansion of AsA-GSH cycle genes

Gene duplication through whole-genome, segmental, or tandem events is a major driver of plant genome evolution [46][47]. In plants like *A. sativum*, *Zea mays*, and *Phyllostachys edulis*, WGD has led to significant gene expansions in *APX*, *MDHAR*, and *GR* gene families, enabling plants to manage both secondary metabolism and oxidative stress (Fig. 1) (Supplementary Table S1). Plants in extreme environments, such as conifers (*Pinus taeda*) and desert species (*Welwitschia mirabilis*), exhibit higher gene redundancy, likely aiding in ROS detoxification under stress. In contrast, plants in stable environments like chlorophytes (*Dunaliella salina*) and bryophytes (*Marchantia polymorpha*) show more conservative gene distributions, suggesting less evolutionary pressure for gene duplication. In angiosperms, gene expansions are linked to specific ecological adaptations, such as in *Pistacia vera*, where increased *MDHAR* genes support nitrogen fixation, and *Camellia chekiangoleosa*, whose expanded *GR* genes aid in secondary metabolism.

Synteny analysis of 2424 AsA-GSH cycle genes revealed extensive networks across plant lineages, with homologous gene pairs forming major clusters (Fig. 2D). The *APX* gene family has four main clusters, with distinct subgroups (APX-1/2, APX-3/5, APX-4/6, and stAPX) demonstrating conserved functions. Notably, APX-1/2 subgroup genes presented weak synteny with other clusters, supporting their distinct evolutionary origin. As the largest synteny cluster, APX-3/5 subgroup displays remarkable conservation across plant lineages, highlighting its essential contribution to oxidative stress responses. Bridge genes connecting different APX subgroups may represent intermediate stages in the family’s evolution, highlighting the dynamic nature of gene diversification in plants.

### Characteristics and functional analysis of grapevine AsA-GSH cycle genes

Given that gene family expansions often underlie complex trait evolution, grapevine with its well-characterized genome and traits closely linked to antioxidant metabolism serves as an ideal model to examine the functional diversity of AsA-GSH cycle genes. Based on 16 published grapevine pan-genome data analysis [48,49], we observed that wild North American species such as *V. riparia* (26 genes), *V. monticola* (21 genes), and *V. berlanderi* (20 genes) had larger gene families compared to the domesticated *V. vinifera* (16 genes). The significant expansion of *MDHAR* and *GR* genes in the wild species *V. vinifera* compared with the cultivated species *V. vinifera* indicates that *V. vinifera* may have experienced gene loss during domestication, resulting in a significant reduction in the diversity of its antioxidant-related genes. In contrast, wild *Vitis* species, often grown in areas with greater environmental stress, retain more antioxidant genes in their genomes, which helps improve their ability to cope with oxidative stress and other environmental challenges [50].

A total of 16 AsA-GSH cycle genes were identified in the cultivated grapevine species *V. vinifera*, including eight *VvAPXs*, three *VvDHARs*, three *VvMDHARs*, and two *VvGRs*, with phylogenetic analysis revealing distinct biological roles based on subcellular compartmentalization (Fig. 3A). Gene structure analysis indicated functional diversification, and promoter regions contained 234 CREs, primarily linked to hormone regulation, growth, and stress responses (Fig. 3B). In total, 519 TFs from 30 families were identified, with MIKC_MADS, MYB, NAC, and ERF being the most abundant, indicating a complex regulatory network. (Fig. 3C–E). Gene duplication and collinearity analyses suggested evolutionary conservation across species, with segmental duplications and purifying selection being predominant, while certain gene pairs exhibited signs of functional divergence (Fig. 4A–C). To investigate the functional relevance of AsA-GSH cycle genes in grapevine development, we analyzed their expression across 54 tissues and organs using a global transcriptome atlas (Fig. 5A) (Supplementary Table S8). Several genes, including *VvAPX5/7/8*, *VvDHAR1*, *VvMDHAR1/2/3*, and *VvGR1/2*, exhibited broad expression across all developmental stages, suggesting constitutive roles in growth and redox homeostasis. Specific spatiotemporal patterns were also clearly observed. *VvAPX1/3/5* were highly expressed during fruit ripening, while *VvDHAR1* and *VvGR1* were highly expressed in young leaves and seeds, and *VvAPX6/7* were highly expressed in mature woody tissues. Notably, *VvMDHAR1* and *VvAPX7* showed root-specific expression, implying roles in belowground stress responses. These transcriptomic findings were validated by RT-qPCR (Fig. 5B–C).

Under hormone treatments (Fig. 6A), ABA elicited the strongest and most rapid induction, especially within 1–6 h, notably in *VvAPX1/6/7*, *VvDHAR1*, *VvMDHAR2*, and *VvGR2*, suggesting their involvement in ABA-mediated stress signaling. SA also strongly upregulated *VvAPX1/2* and *VvGR1*, linking them to biotic stress responses. MeJA triggered the expression of *VvAPX5*, *VvMDHAR2*, and *VvGR2*, while GA induced milder responses, mainly associated with growth regulation. A predicted network of 1149 TFs, including MYB, bHLH, ERF, NAC, and WRKY, may regulate these genes during stress, development, and hormone signaling (Fig. 6E). RT-qPCR validation further supported the differential expression of *VvAPX1*/*6*/*7*, *VvDHAR1*, *VvMDHAR2*, and *VvGR2* under various hormone treatments, particularly ABA and SA, emphasizing their potential role in stress resistance and defense mechanisms (Fig. 6I).

Grapevine downy mildew, caused by *P. viticola*, is a significant threat to viticulture. Our study tracked pathogen colonization in ‘Thompson Seedless’ grape leaves (Fig. 7A–B). Simultaneously, we examined the involvement of AsA-GSH cycle genes in the grapevine’s reaction to the pathogen. Twelve genes were differentially expressed during infection, with *VvAPX1*/*5*/*7* upregulated at 12 and 24 h, and *VvDHAR1*/*3* strongly induced (Fig. 7C). Cluster analysis revealed temporal expression patterns, with Cluster 5 genes upregulated at 24 h and Cluster 2 genes showing early downregulation followed by recovery (Fig. 7D). GO and KEGG analyses linked these genes to stress response and DNA repair (Fig. 7E–F). WGCNA and TF network analysis identified coordinated regulation involving bHLH, MYB, and NAC TFs (Fig. 7H–J). Expression profiling showed time-dependent expression, with early upregulation of *VvAPX1*/*5*/*6*/*7* indicating immediate immune responses, while later-stage genes like *VvMDHAR2* were linked to recovery mechanisms (Fig. 7K).

Abiotic stress analysis revealed that the majority of genes exhibited varied responses. Cold stress induced nearly all genes strongly, whereas relatively lower expression levels were induced by heat stress. Both salt and drought stresses quickly activated a number of genes at the early stages to boost ROS scavenging (Fig. 7L). To assess their oxygen species (ROS) scavenging ability, transient expression of these proteins was performed, and western blot analysis confirmed successful protein expression (Fig. 8B). The enzyme activity assays showed significant reductions in H₂O₂ and O₂·^−^ in the experimental groups compared to the GFP control, particularly in VvAPX8, VvDHAR1, VvMDHAR2, and VvGR2. Moreover, overexpression of these genes under abiotic stresses (cold, heat, salt, and drought) resulted in decreased ROS and MDA levels, while T-AOC was significantly increased, indicating their potential to alleviate oxidative stress and enhance antioxidant defenses in plants (Fig. 8C–D).

### Potential application of AsA-GSH cycle genes in grapevine breeding

Our pan-genomic analysis revealed a striking divergence in the composition and copy number of AsA-GSH genes across grapevine species originating from different geographic regions. North American wild grape species such as *V. riparia*, *V. monticola*, and *V. berlanderi* exhibit greater numbers of *APXs*, *MDHARs*, and *GRs* than the cultivated Eurasian species *V. vinifera*, suggesting a possible evolutionary adaptation to stresses. These stress-tolerant genotypes represent a rich reservoir of allelic diversity that can be harnessed for trait introgression into elite cultivars[51–53]. *VvAPX6*/*7*/*8*, *VvDHAR1*, *VvMDHAR2*, and *VvGR2* identified here as key components in ROS detoxification, have demonstrated strong transcriptional and functional responses under stress and can be stable transformation or promoter engineering. Previous studies have already shown that the WRKY TF *TTC4* is significantly upregulated under heat stress and can directly activate *APX3* (*Vitvi03g00373*), thereby enhancing thermotolerance in grapevine [54]. Overexpression of *VvAPX1*(*Vitvi014747*) enhances resistance to the downy mildew pathogen *P. viticola* in both grapevine and *N. benthamiana* via increased APX activity [55]. These findings provide a foundation for the targeted application of these genes in stress-resilient grapevine breeding. Future studies could focus on transforming multiple favorable alleles from wild relatives into susceptible grapevine lines or manipulating core network regulators to optimize redox balance under stress conditions.

## Materials and methods

### Plant genomes collection

We assembled a thorough dataset of 127 plant genomes, covering a wide variety of taxonomic groups. The dataset includes 113 angiosperms, 2 bryophytes, 2 charophytes, 2 chlorophytes, 3 ferns, and 5 gymnosperms (Supplementary Table S1), which were mainly obtained from the CNGBdb (https://db.cngb.org/) [56], Phytozome (https://phytozome-next.jgi.doe.gov/) [57], TreeGenes (https://treegenesdb.org/) [58], FernBase (https://fernbase.org/) [59], HortGenome Search Engine (http://hort.moilab.net) [60], GrapeGenomics (https://www.grapegenomics.com/index.php), and Ensembl Plants (http://plants.ensembl.org/index.html).

### Deep learning to identify AsA-GSH cycle genes

The DeepGOPlus [61] is an advanced model integrating deep learning methods with sequence homology analysis to predict protein molecular features. By applying a prediction threshold of 0.5, the system analyzed genomic-scale datasets from 127 plant species. Homology alignment and hidden Markov model (HMM) were used to predict AsA-GSH cycle genes in the genomes of various species. For homology alignment, orthologous proteins involved in the AsA-GSH cycle served as evolutionary anchors for identifying conserved candidate genes in each species through cross-species BLASTP alignment with Arabidopsis proteome (*E-*value ≤ 1e^−5^). Candidate protein sequences were retrieved and analyzed through the SMART database [62], with *E-*value ≤ 1e^−5^. For hidden Markov model retrieval, two methods were used. One method was to construct HMM with AsA-GSH cycle gene proteins of Arabidopsis, and then search for proteins in the genomes of various species with *E-*value ≤ 1e^−5^; while the other one was to use the characteristic domain APX (PF00141), DHAR (PF02798), MDHAR (PF07992), and GR (PF00462) of AsA-GSH cycle gene protein in the Pfam database [63] to search for proteins with *E-*value ≤ 1e^−5^ in the genomes of various species. All candidate AsA-GSH cycle genes were evaluated using the InterProScan database (http://www.ebi.ac.uk/interpro/) for the presence of conserved heme peroxidase domain (IPR002016), glutathione S-transferase (GST) C-terminal domain (cd03201), MDHAR 3-like, C-terminal domain (IPR048618), and glutathione reductase domain (IPR006324). The AsA-GSH cycle genes were renamed based on their chromosomal loci. Their physicochemical properties were analyzed using ExPASy’s ProtParam tool [64]. Subcellular localization predictions were conducted via the Cell-PLoc 2.0 [65].

### Phylogenetic analysis of the AsA-GSH cycle genes

Phylogenetic analysis was conducted using amino acid sequences of four core enzymes in the AsA-GSH cycle, such as APXs, DHARs, MDHARs, and GRs to elucidate their evolutionary relationships. Multiple sequence alignment of the protein sequences was performed using MAFFT (v7.526) [66] and were then trimmed by TrimAl (v2.0) [67]. Phylogenetic trees were inferred using ML as implemented in IQ-TREE 2 (v2.3.4) [68] software with best-fit model chosen using WAG+G4 [69] (AsA-GSH cycle genes tree), JTT + R9 [70] (APX tree), JTT + R7 [71] (DHAR/GR tree), and Q.plant+R7 [71] (MDHAR tree) models. The robustness of phylogenetic branches was quantified through 1000 iterations of bootstrap analysis, while genetic divergence measurements derived from pairwise sequence comparisons served as the basis for branch length estimation. Missing nucleotide positions were processed using selective gap exclusion methodology. The phylogenetic trees were visualized using the online tool Evolview v3 [72].

### Chromosome location and haplotype-to-haplotype comparison of collinearity

Synteny and collinearity of gene pairs between grapevine’s genomes were identified with JCVI [73]. Syntenic gene pairs between haplotypes were identified using JCVI with a cutoff of −cscore = 0.99 to generate one-to-one pairs. The mapping of all grapevine AsA-GSH cycle genes to their respective chromosomes was performed in Circos [74].The WGDI [75] was utilized to visualize the synteny of orthologous AsA-GSH cycle genes across grapevine and various other species. Divergence times (MYA) for the grapevine AsA-GSH cycle genes were estimated using the following formula: Time = *K*s/2λ, where λ is set at 6.5 × 10^−9^ [76].

### Gene structure, conserved motifs, and CREs analysis

The intron-exon structures of the grapevine AsA-GSH cycle genes were derived from the GFF annotation files. Protein sequence analysis of Multiple Em for Motif Elicitation (MEME v5.5.8) online program (https://meme-suite.org/meme/) was used to identify the conservative motif in AsA-GSH cycle gene proteins. The optimization settings include an arbitrary number of repetitions, with a maximum limit of 10 motifs. The promoter regions, defined as 2000 bp upstream of the ATG codon of grapevine AsA-GSH cycle genes, were extracted using TBtools (v2.12) [77] aligned against the complete genomic DNA sequences of grapevine. Putative promoter regions of grapevine AsA-GSH cycle genes in grapevine CREs were predicted using the PlantCare database [78].

### Structural modeling analysis

The structure prediction of grapevine AsA-GSH cycle gene proteins by AlphaFold2 were carried out using the Alphafold Google Colab server with the MSA_method [79]. The AlphaFold2 algorithm assigns residue-specific confidence assessments (pLDDT) spanning 0–100, with protein domains exhibiting pLDDT values <50 predicted to lack ordered conformation in isolation. The structural graphics and analyses were performed with PyMOL (https://pymol.org/2/).

### TFs regulatory network analysis

Transcriptional regulatory networks governing grapevine AsA-GSH cycle genes were interrogated using the PlantTFDB [40], with CREs prediction constrained to proximal 2000 bp promoter regions under rigorous statistical filtering (*P*-value ≤1 × 10^−7^). Cross-validation with Arabidopsis orthologues established evolutionary conservation of these regulatory architectures. The resulting TFs were visualized in Cytoscape (v3.9.1) [80], and a word cloud was generated using the ggplot2 package [81] in R.

### Plant materials and multiple stress treatment

The grapevines situated within Center for Viticulture and Enology, Shanghai Jiao Tong University (Minhang District, Shanghai City, China), serves as the focal site for this study. *V. vinifera* ‘Cabernet Sauvignon’ grapevines were initially propagated via tissue culture on half-strength Murashige and Skoog (1/2 MS) medium supplemented with 0.15 mg l^−1^ indole-3-butyric acid for 5 weeks. The stabilized growing plantlets were then transplanted into pots containing a peat-vermiculite mixture (1:1, v/v) and grown for additional 5 weeks under controlled conditions. Grapevines and *N. benthamiana* were cultivated in a greenhouse environment under precisely controlled conditions, with the daytime temperature and nighttime temperature maintained at approximately 25 ± 2°C. The photoperiod was consistently set at 16 h of light provided by cool-white fluorescent lamps, delivering an intensity of around 150 μmol m^−2^ s^−1^ at plant canopy level, followed by 8 h of darkness. For hormone treatment, leaves were sprayed with 200 μM of abscisic acid (ABA), gibberellic acid (GA), salicylic acid (SA), and methyl jasmonate (MeJA) in an aqueous medium. Samples were taken at 0 (CK), 1, 3, 6, 12, and 24 h after treatment. For biotic treatment, leaf discs were inoculated with *P. viticola* ‘Pv18001’ using 30 μl of a spore suspension at a concentration of 10^5^ spores/ml. Samples were taken at 0, 3, 6, 12, 24, and 48 h after inoculation. For abiotic treatments, the leaves were immersed in 150 mM NaCl and 150 mM mannitol to simulate salt and drought stress, respectively. For cold and heat treatments, the plantlets were placed in growth chambers set to 4 and 45°C, respectively. The samples were harvested at 0 (CK), 1, 3, 6, 12, and 24 h following treatment. Three biological replicates were performed for each treatment.

### RNA extraction and RT-qPCR analysis

The third to fifth fully expanded leaves from the top of shoots were collected, and RNA extraction was performed using the modified CTAB method as previously reported [82]. cDNA synthesis was carried out using the ThermoScript RT-PCR kit (Yeasen, 11201ES03, Shanghai, China). The BIO-RAD CFX Manager was used to analyze relative expression levels. Primer sequences for RT-qPCR are listed in Supplementary Table S13. Gene expression assays were performed in triplicate with biological replicates, and fold changes were determined using the 2^−ΔΔCT^ method.

### Transcriptome (RNA-seq) analysis

The quality of raw RNA-seq reads was filtered and mapped with fastp (v0.23.4) [83], then mapped to the PN40024’s genome (T2T.v5) using HISAT2 (v2.2.1) [84] and Samtools (v1.2.0) [85]. The Mfuzz package [86] was used to analyze gene expression patterns in response to hormone and *P. viticola* treatments, followed by identification of clustering groups. The WGCNA package (v1.69) [87] was used to perform WGCNA analysis. The edge weight (ranging from 0 to 1) between two genes in the co-expression network was calculated based on their topological overlap measure, and the networks were visualized with Cytoscape (v3.9.1) [80]. In addition, publicly available gene expression profiles from microarray data, representing various grapevine organs and developmental stages, were retrieved from the NCBI Gene Expression Omnibus (GEO) under accession number GSE36128 [41]. These datasets were utilized exclusively for tissue-specific expression profiling of AsA-GSH cycle genes.

### Histochemical staining analysis

Leaf discs were stained with trypan blue and aniline blue solution as previously reported [88]. To perform trypan blue staining, the samples were decolorized in a 2.5 g/ml chloral hydrate solution until they became colorless and transparent, after which images of the leaf samples were taken. For aniline blue staining, the samples were placed in 1 M KOH and autoclaved at 121°C for 10 min, then cooled to room temperature. After washing with distilled water, the samples were stained for 10 min with 0.05% aniline blue (dissolved in 67 mM KH₂PO₄, pH 9.0–10.0). Hyphal development and infection were observed using a fluorescence microscope (Olympus BX-51) under blue/violet light (excitation 400–440 nm, emission 475 nm).

### Transient expression in *N. benthamiana* via agrobacterium infiltration

To co-express the plasmids and control constructs, agroinfiltration was carried out in *N. benthamiana* leaves. The coding sequences of six selected grapevine AsA-GSH cycle genes (*VvAPX6*/*7*/*8*, *VvDHAR1*, *VvMDHAR2*, and *VvGR2*) were cloned into the binary vector pCAMBIA1300-GFP under the control of the CaMV 35S promoter [89]. PT:RFP [90] was used as an indicator of the plastidial subcellular localization of VvGR2. The recombinant constructs and empty GFP control vector were introduced into *Agrobacterium* strain GV3101 via electroporation. *Agrobacterium* strains containing the binary vectors were grown in liquid Luria-Bertani medium supplemented with the appropriate antibiotics at 28°C at 200 rpm agitation. Bacterial cells were harvested by centrifugation and subsequently resuspended in an infiltration buffer (10 mM MgCl₂, 10 mM MES pH 5.8, 200 μM acetosyringone). The resulting suspension was infiltrated into *N. benthamiana* leaves using a needleless syringe.

As previously described [91,92], transient expression assays were performed in tobacco to assess the potential functions of *VvAPX6*/*7*/*8*, *VvDHAR1*, *VvMDHAR2*, and *VvGR2*. Tobacco plants were infiltrated with the empty vector (control) or with constructs harboring *VvAPX6*/*7*/*8*, *VvDHAR1*, *VvMDHAR2*, and *VvGR2*. Following transformation, all plants were subjected to low-temperature (4°C), high-temperature (42°C), high salinity (300 mM NaCl), and drought (300 mM mannitol) stress treatments for 12 h in three biological repeats.

### Protein extraction and western blot

Total protein extraction was carried out as previously reported [93]. Plant tissues were immediately frozen in liquid nitrogen and subsequently homogenized in a prechilled protein extraction buffer. After a 10-min incubation on ice, samples were centrifuged at 4°C for 10 min to obtain the supernatant. The supernatant was mixed with 5× SDS loading buffer and boiled at 100°C for 5 min. Proteins were separated using 10% SDS-PAGE and detected by immunoblotting with the specified antibodies.

### Measurement of physiological indices

The concentrations of H₂O₂ (Solarbio-BC3590), O₂˙^−^ (Solarbio-BC1290), T-AOC (Solarbio-BC1315), MDA (Solarbio-BC0025), APX (Solarbio-BC0220), DHAR (Solarbio-BC0660), MDHAR (Solarbio-BC0650), and GR (Solarbio-BC1160) were determined using commercial assay kits according to the manufacturer’s protocols (Solarbio Technology Co., Ltd, Beijing, China).

### Quantification and statistical analysis

Statistical analysis was performed using GraphPad software (v10.2.3). Specifications of tests exploited and sample size for each experiment are mentioned in the figure legends. Data are presented as the standard error of the mean. Differences were analyzed by the student’s *t*-test and one-way ANOVA as indicated in the figure legends. Asterisk (*) indicates ^*^P <0.05, ^**^P <0.01, and ^***^P <0.001 against each control group, respectively.

## Supplementary Material

Web_Material_uhaf247

## Data Availability

The supporting data for this study can be found in the Supplementary materials. RNA-seq data have been deposited in CNGB (CNP0007884) and NCBI (PRJNA1157543).
