# Correction to: Evolutionary analysis of ascorbate-glutathione cycle genes across green plants with lineage-specific profiling in grapevine (*Vitis vinifera* L.)

**DOI:** 10.1093/hr/uhag257

**Published:** 2025-07-06

**Authors:** Jianxiang Liang, Menghao Xu, Bohan Yang, Jiaqi Liu, Zhizhuo Xu, Xiukun Yao, Jiang Lu, Peining Fu

**Affiliations:** Center for Viticulture and Enology, School of Agriculture and Biology, Shanghai Jiao Tong University, Shanghai 200240, China; Center for Viticulture and Enology, School of Agriculture and Biology, Shanghai Jiao Tong University, Shanghai 200240, China; Center for Viticulture and Enology, School of Agriculture and Biology, Shanghai Jiao Tong University, Shanghai 200240, China; Center for Viticulture and Enology, School of Agriculture and Biology, Shanghai Jiao Tong University, Shanghai 200240, China; Center for Viticulture and Enology, School of Agriculture and Biology, Shanghai Jiao Tong University, Shanghai 200240, China; Center for Viticulture and Enology, School of Agriculture and Biology, Shanghai Jiao Tong University, Shanghai 200240, China; Plant Conservation & Breeding Technology Center, Guizhou Key Laboratory of Agricultural Biotechnology, Guizhou Academy of Agricultural Sciences, Guiyang, Guizhou 550006, China; Plant Conservation & Breeding Technology Center, Guizhou Key Laboratory of Agricultural Biotechnology, Guizhou Academy of Agricultural Sciences, Guiyang, Guizhou 550006, China

This is a correction to: Jianxiang Liang, Menghao Xu, Bohan Yang, Jiaqi Liu, Zhizhuo Xu, Xiukun Yao, Jiang Lu, Peining Fu, Evolutionary analysis of ascorbate-glutathione cycle genes across green plants with lineage-specific profiling in grapevine (*Vitis vinifera* L.), Horticulture Research, Volume 13, Issue 1, January 2026, uhaf247, https://doi.org/10.1093/hr/uhaf247

In the originally published version of this manuscript, the collinearity plot of V. vinifera vs V. rotundifolia in Figure 4B was inadvertently displayed as a duplicate of the V. vinifera vs V. amurensis plot, due to an error during the file assembly process.

Figure 4 has now been updated with the corrected version.

**Figure 4:**
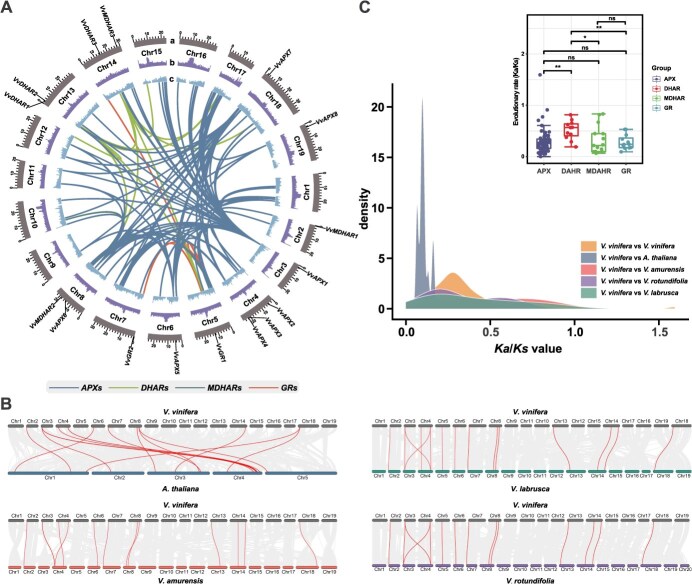
Chromosomal distribution and gene synteny.(A) Distribution and collinearity of AsA-GSH cycle genes in the grapevine genome. Panels:(a) 19 chromosomes with mega base units;(b) GC content;(c) Gene density.(B) Synteny analysis of AsA-GSH cycle genes in European (V. *vinifera*), East Asian (V. *amurensis*), Muscadinia (V. *rotundifolia*), and American (V. *labrusca*) species, with red lines highlighting syntenic gene pairs.(C) Histogram of *Ka/Ks* ratios for AsA-GSH cyclegenes

