# Supplementary material for: Evolutionary analysis of ascorbate-glutathione cycle genes across green plants with lineage-specific profiling in grapevine (Vitis vinifera L.)
Source: Hortic Res. 2025 Sep 19;13(1):uhaf247. doi: 10.1093/hr/uhaf247 (PMC12854088; doi:10.1093/hr/uhaf247)
Supplement: Web_Material_uhaf247 [file web_material_uhaf247.zip › Supplementary Figure.pdf]

Supplemental figures for

Evolutionary analysis of ascorbate-glutathione cycle genes across green plants with lineage-specific profiling in grapevine (*Vitis vinifera* L.)

Jianxiang Liang, Menghao Xu, Bohan Yang, Jiaqi Liu, Zhizhuo Xu, Xiukun Yao, Jiang Lu*, Peining Fu*

*** Correspondence authors:**

Jiang Lu: jiang.lu@sjtu.edu.cn

Peining Fu: fupeining@sjtu.edu.cn

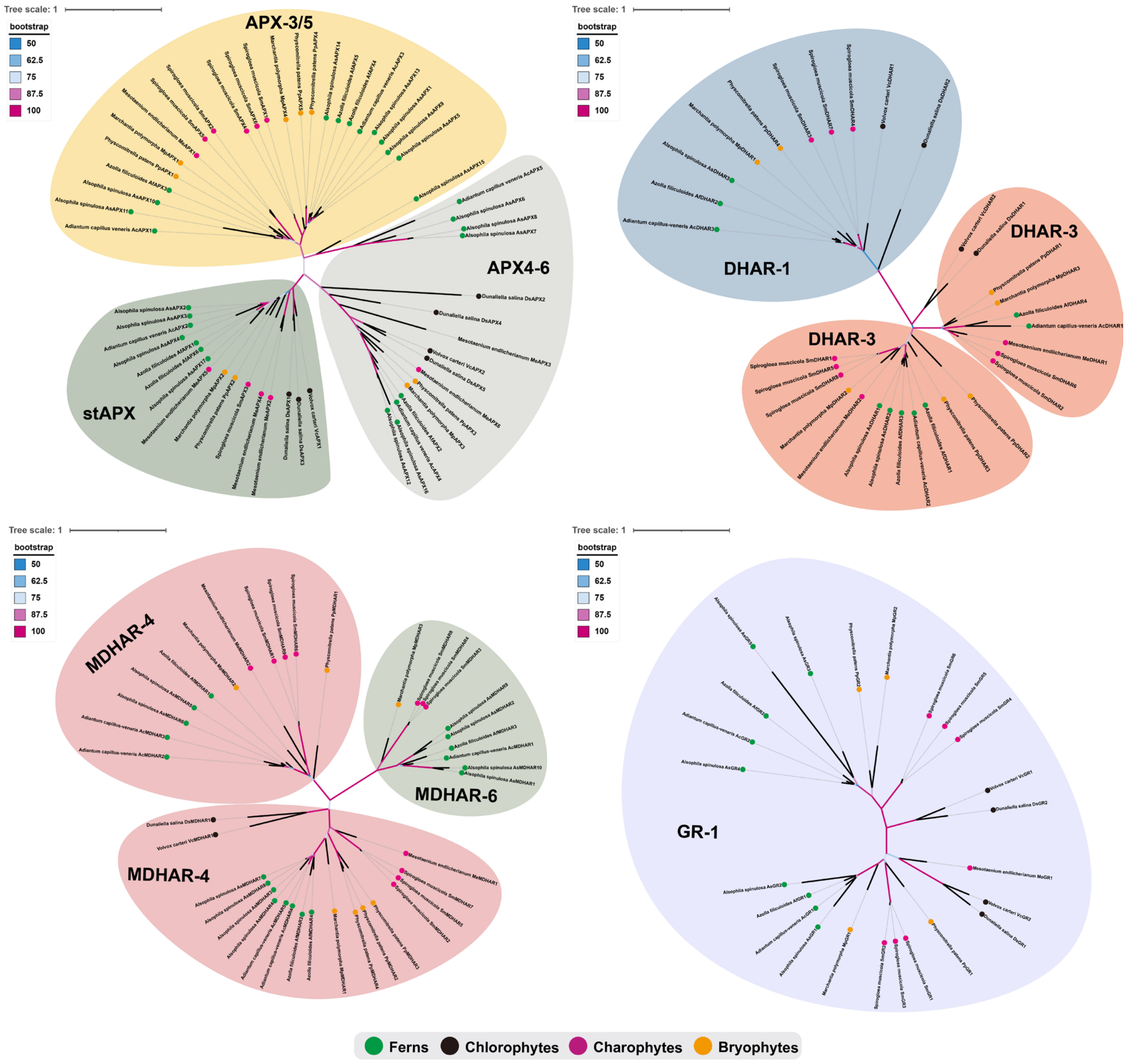

Figure S1. Phylogeny of the *APX*, *DHAR*, *MDHAR*, and *GR* gene family of the water-land transition zone. Maximum likelihood (ML) phylogenetic trees were constructed using model-specific substitution schemes: WAG+R4 for APX tree, Q.pfam+I+G4 for DHAR tree, and Q.pfam+G4 for MDHAR and GR trees.

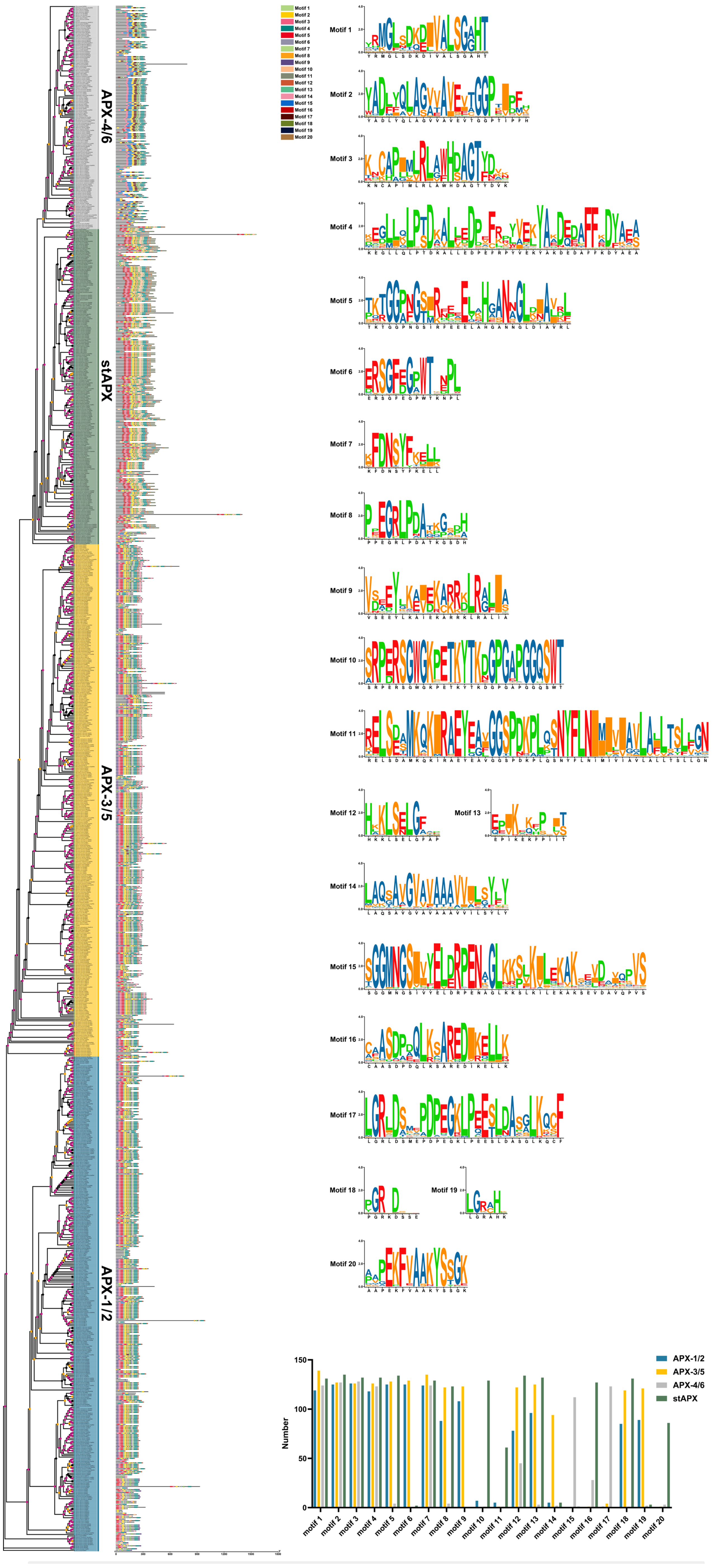

Figure S2. Characteristic sequence motif fingerprints of *APX* gene family. Twenty conserved motifs were detected in the sequences and visualized using different colors (Motif 1-20) to indicate their positions and diversity.

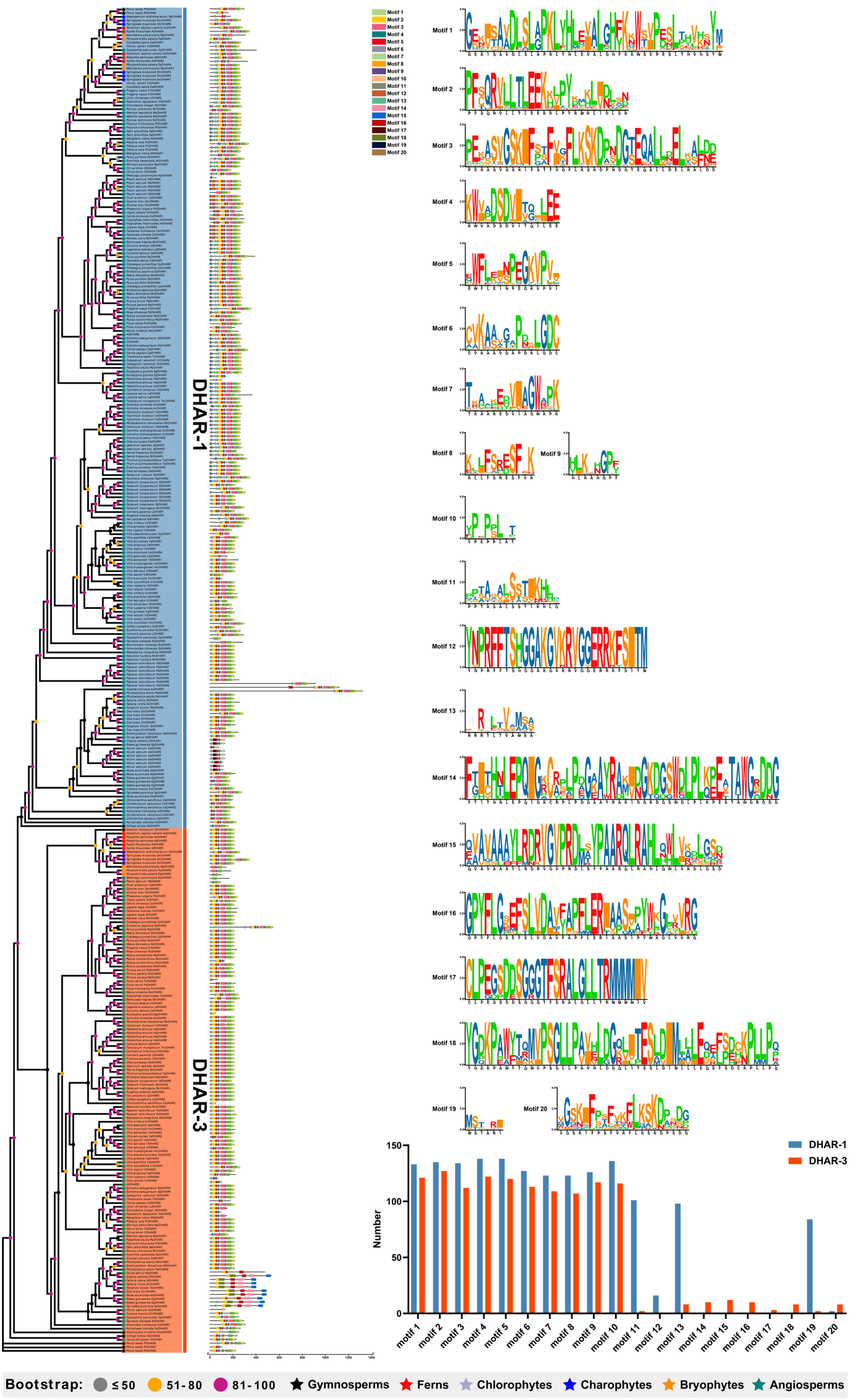

Figure S3. Characteristic sequence motif fingerprints of *DHAR* gene family. Twenty conserved motifs were detected in the sequences and visualized using different colors (Motif 1-20) to indicate their positions and diversity.

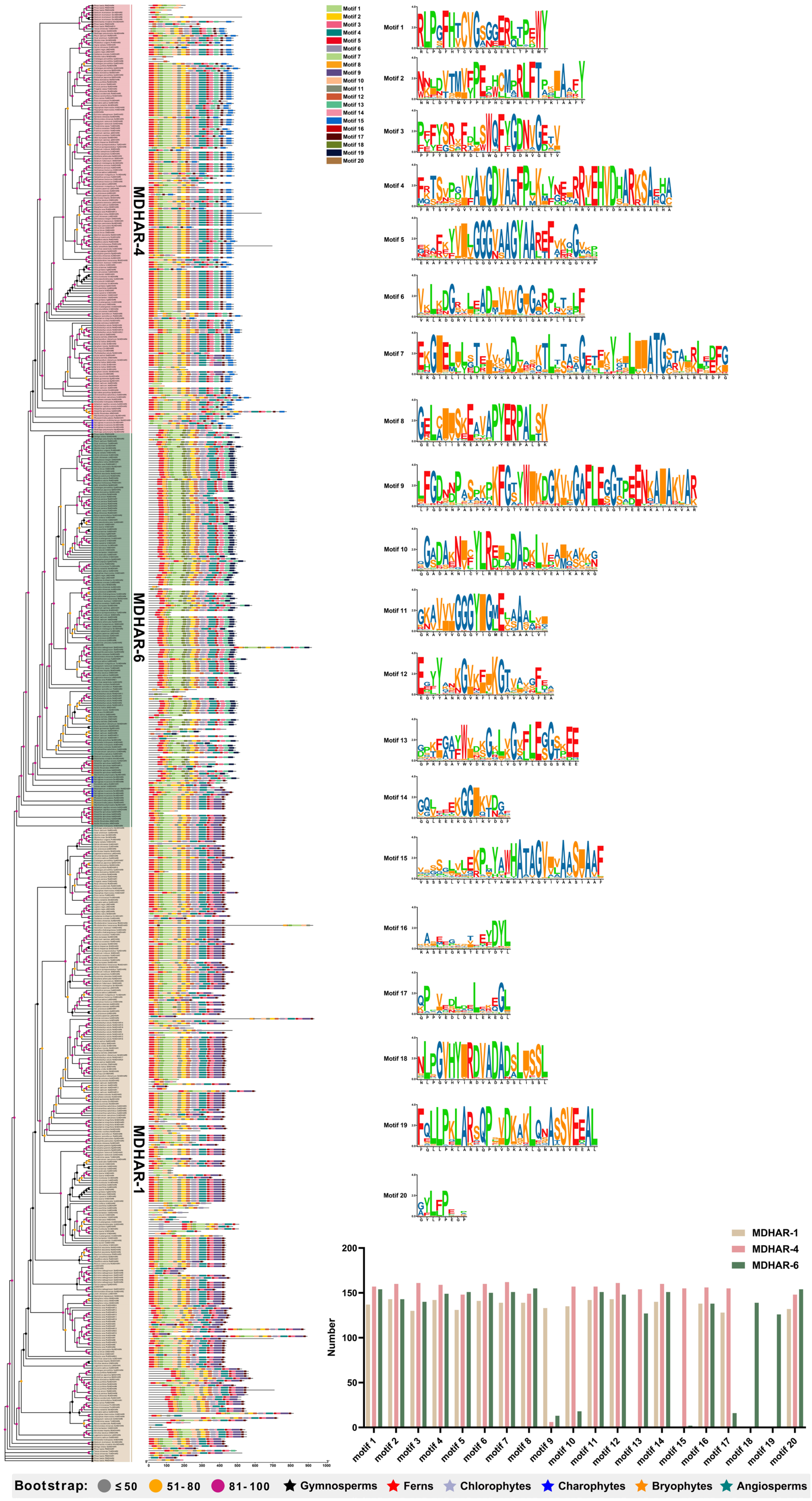

Figure S4. Characteristic sequence motif fingerprints of *MDHAR* gene family. Twenty conserved motifs were detected in the sequences and visualized using different colors (Motif 1-20) to indicate their positions and diversity.

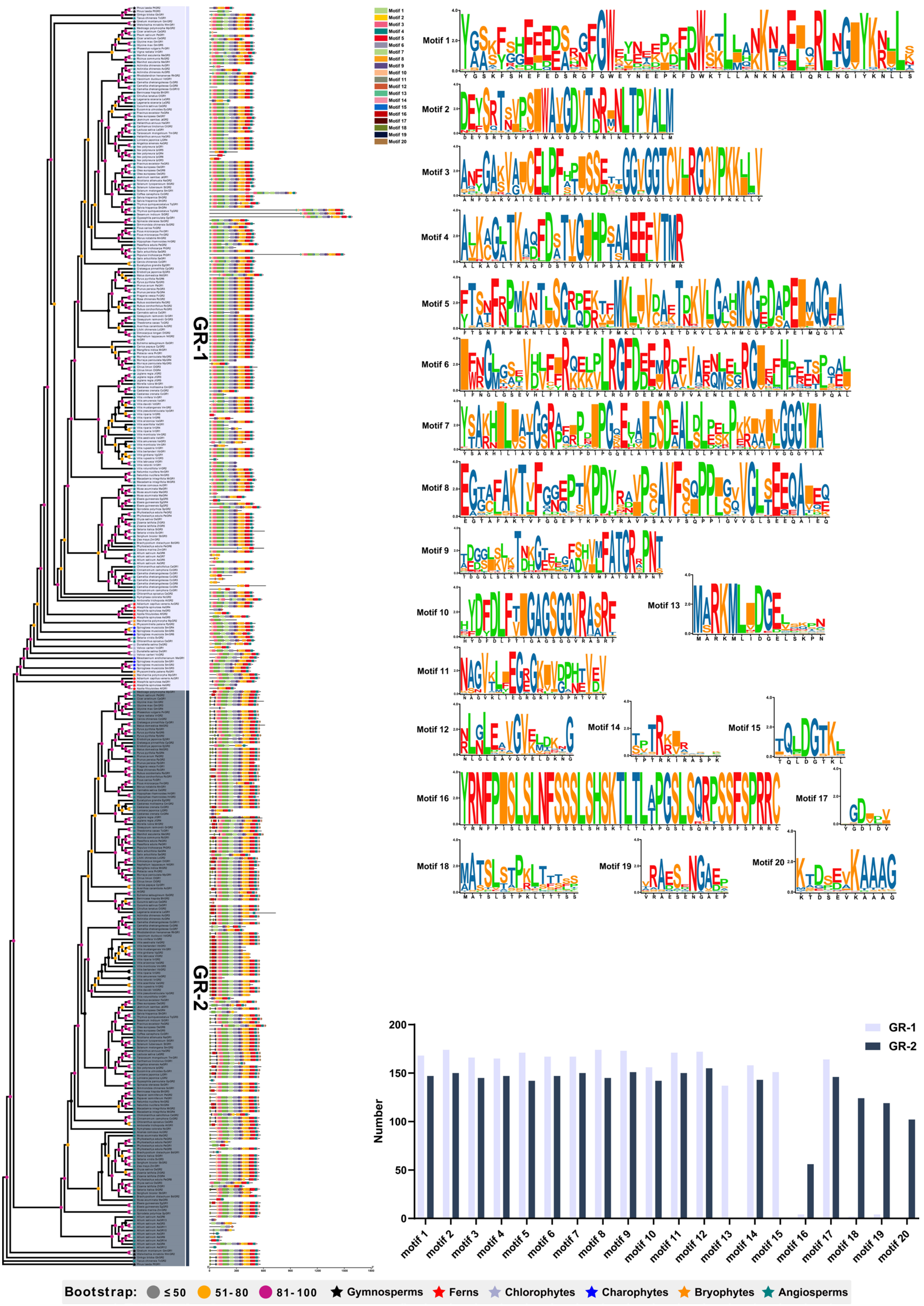

Figure S5. Characteristic sequence motif fingerprints of *GR* gene family. Twenty conserved motifs were detected in the sequences and visualized using different colors (Motif 1-20) to indicate their positions and diversity.

Note: To discover potential novel conserved motifs in AsA-GSH cycle proteins, we utilized the MEME tool for local multiple motif induction to analyze the protein sequences of the candidate AsA-GSH cycle genes. We identified an additional 9 to 18 conserved motifs within the amino acid domains of the AsA-GSH cycle genes, with the variation likely arising from differences in gene structure across species or subfamilies. This range reflects the diversity of the AsA-GSH cycle gene family, where certain genes may present a higher or lower number of conserved motifs due to functional specialization or evolutionary divergence. The composition of these new specific continuous motifs spans about 280, 210, 410, and 490 amino acids in length and can be distributed and defined as APX, DHAR, MDHAR and GR domains (**Fig. S2-5**). Multiple sequence alignment (MSA) of APX, DHAR, MDHAR and GR proteins from several representative species was performed, and conserved APX, DHAR, MDHAR and GR domain regions were selected to construct a training dataset for the APX, DHAR, MDHAR and GR domain seed models. By testing the ASA-GSH domain seed model with various protein datasets, and the findings indicated that domain seed model can be employed as a query to facilitate the identification of ASA-GSH cycle genes in other species.

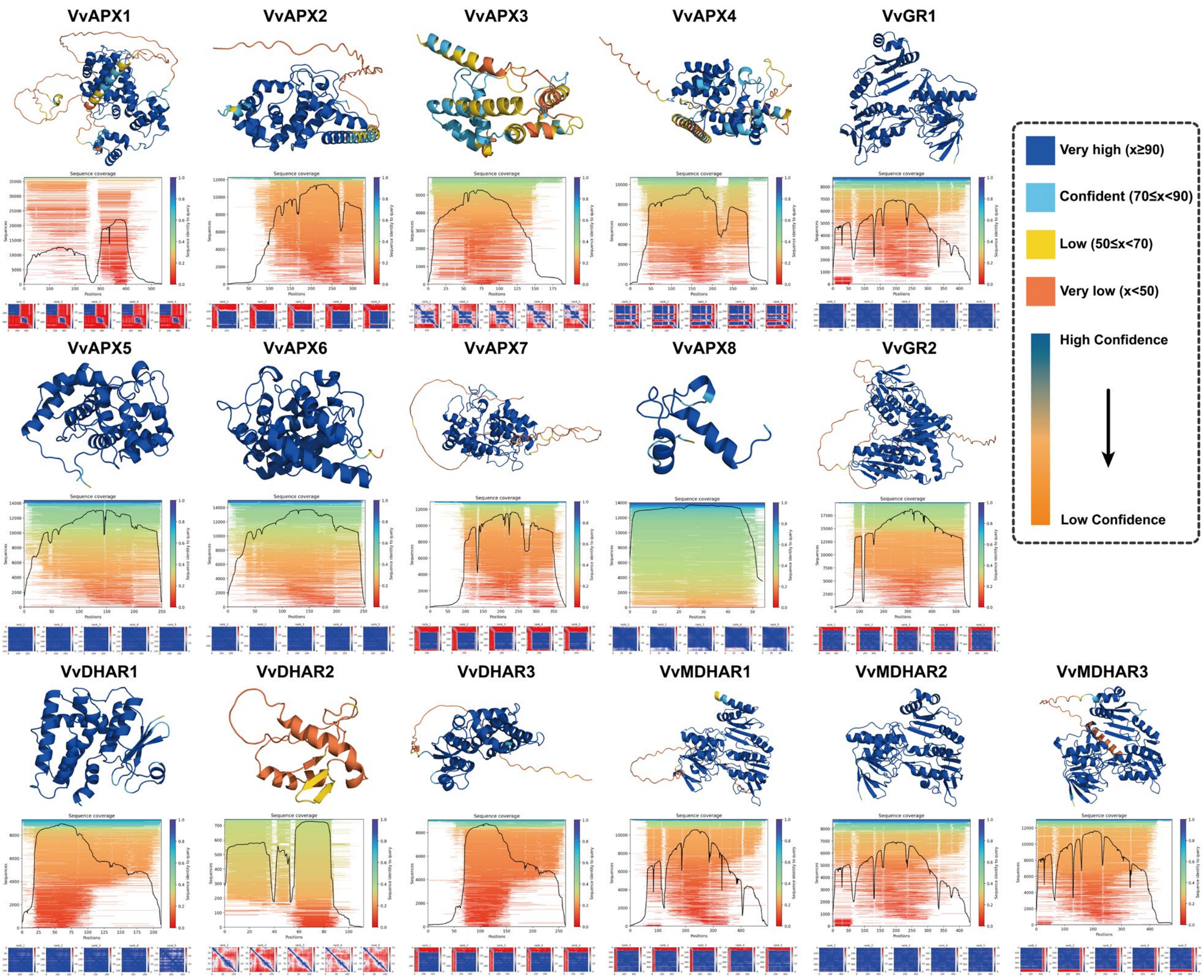

Figure S6. Structural analysis of the grapevine AsA-GSH cycle protein. Residues are color-coded according to their predicted local quality values, reflecting the confidence level of the structural model at each position.

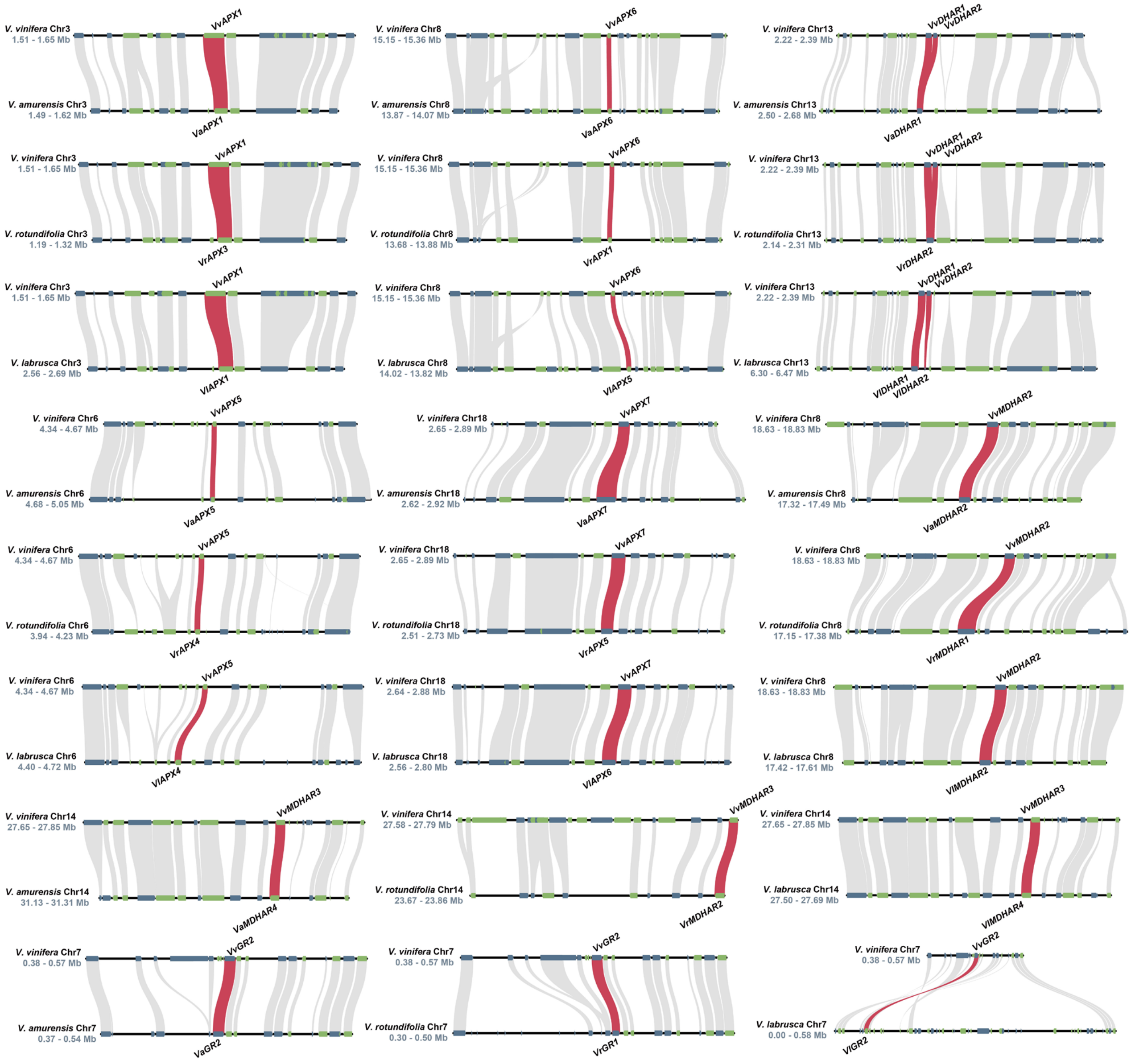

Figure S7. AsA-GSH cycle genes microsynteny analysis of *V. vinifera*, *V. amurensis*, *V. labrusca*, and *V. rotundifolia*. Gray lines in the background indicate collinear blocks in grapevine and other plant genomes, while red lines highlight syntenic gene pairs.
